# Baseline emotional state influences on the response to animated short films: A randomized online experiment

**DOI:** 10.3389/fpsyg.2022.1009429

**Published:** 2022-12-15

**Authors:** Juliana Gioia Negrão, Paulo Rodrigo Bazán, Raymundo Machado de Azevedo Neto, Shirley Silva Lacerda, Eve Ekman, Elisa Harumi Kozasa

**Affiliations:** ^1^Hospital Israelita Albert Einstein, São Paulo, Brazil; ^2^Greater Good Science Center, Department of Psychology, University of California, Berkeley, Berkeley, CA, United States

**Keywords:** emotion, animated short film, refractory period, mental health, emotion regulation, COVID-19

## Abstract

**Introduction:**

Considering the relevance of the emotional state, it is necessary to understand how daily stimuli can modulate the emotions. Animated short films are common stimuli, but it is unknown how they can modulate the emotional state. The study aimed to evaluate: how participants’ emotional state changed after watching animated short films with positive or negative emotional valence in an online experiment; the relationship between participants’ baseline score on an Emotional Intensity Scale and their potential change in the main emotion after watching the films; and the association between the initial main emotion valence and the potential change in this emotion with participants’ sociodemographic information.

**Methods:**

A sample of 2,269 participants recruited during COVID-19 pandemic were randomly assigned to either watch a negative or positive animated short film.

**Results:**

The results showed that, after watching a film with negative valence, participants were in a more negative emotional state than at baseline and compared with those who watched the film with positive valence. Also, individuals who had a negative baseline emotion and maintained the same emotion after the film had presented higher baseline emotional state scores (more negative emotion) than those who changed their emotions. In addition, the individuals who kept the baseline emotion had an association with age, marital status, level of education and psychiatric disorders, use of medication, and emotional awareness, while the individuals who changed the baseline emotion had an association with age, gender, and following or not social distancing recommendations.

**Conclusion:**

Baseline emotional state may influence the response to animated short films and sociodemographic characteristics are associated with the initial main emotion valence and its potential change in this emotion.

## Introduction

According to Adolphs and Andler (2018), one of the key aspects of emotions is persistence, which means that an emotion can last longer than its cause, having impacts on subsequent behavior, according to our emotional state. Considering the relevance of the emotional state, it is necessary to understand how daily stimuli can modulate the emotions. A common stimulus is the news. Recently, an online experiment evaluated the effects of news with a positive or negative content. Negative news worsened participants’ emotional state, while positive news improved it. These results were found both for the general population (Bazán et al., 2021b) and for healthcare professionals (Bazán et al., 2021a).

Similar results had been found previously by Chuang and Lin (2007) who found effects of news valence on the reported affective emotional state of participants who listened to news while “waiting” to start the experiment. Furthermore, they reported that the news valence could produce impacts on the ways in which individuals perceive and evaluate other’s emotions, indicating that an emotional state may change behavior and perception of others by new stimuli.

Lench et al. (2011) investigated how emotions influence different outcomes ranging from reaction time to prosocial behavior. The results indicated that a small number of core emotions, named discrete emotions, caused changes in cognition, judgment, behavior, and physiology. In addition, an individual current emotional state can be influenced by a new content with emotional valence.

Indeed, few studies revealed that the current emotional state can influence how individuals perceive new information with emotional valence and can create bias (Isen et al., 1978; Bower, 1991; Bouhuys et al., 1995; Schwarz and Clore, 2007; Huntsinger et al., 2010). For example, Bouhuys et al. (1995) examined the relationship between mood and emotional face expressions and concluded that participants of the study when feeling more depressed perceived more rejection/sadness in ambiguous faces (displaying less intensive emotions) and less invitation/happiness in clear faces. Hence, results show a depression-related negative bias in the perception of facial displays. However, aside from the effects that emotions may have on the perception of new stimuli, another aspect of the emotional dynamic is the possible relation between susceptibility to new stimuli and the prior emotional state. Currently, there is a gap in the literature regarding this relation between the emotional variation induced by a stimulus and the baseline emotional state. A theoretical construct has been proposed in which intense emotions could induce a refractory period, in which the person would be less affected by external stimuli that would tend to alter his or her emotional state (Ekman and Cordaro, 2011).

The pandemic offers a unique opportunity to carry out studies about emotions as the emotions of the individuals may be intensified, and their susceptibility to emotional stimuli could be altered. During the COVID-19 pandemic, social distancing measures have revealed significant impacts on emotions and wellbeing, both individually and collectively (Hossain et al., 2020). These impacts have increased significantly after issuance of homestay request and constant reporting of lethality rates (Xin et al., 2020). Furthermore, this isolation combined with the fear of infection, as well as the social and economic burden, harmed people’s mental health (Benke et al., 2020; Bareeqa et al., 2021). It is important to consider that difficulties in emotion regulation, such as stressful situations during the COVID-19-pandemic, can lead to changes in emotional stability and result in mental disorders (Sheppes et al., 2015).

As emotion-inducing stimuli, animated short films were reported with a relatively high degree of ecological validity to elicit emotions, mainly considering the fact that emotions are usually evoked by external dynamic visual and external auditory stimuli (Gross and Levenson, 1995). Using online presentation of such films offers an opportunity to evaluate a larger sample than previous studies during the pandemic. In this context, some participants with intense baseline emotional state may not change their emotions even after watching the animated short films. This could be altered by specific conditions during the pandemic, such as social distancing and work and study activities.

Furthermore, other variables might affect the emotional state and its variation, such as gender (Gabert-Quillen et al., 2015). Age has been suggested to have different effects on emotion perception, affective experience, and emotion regulation (Isaacowitz et al., 2017). The education level seems to be related to different amygdala activity during implicit emotional processing (Demenescu et al., 2014). Therefore, an exploratory analysis of these and other sociodemographic variables could be relevant for a better understanding of the emotional variation.

The present study aimed to evaluate: (1) how participants’ emotional state changes after watching the selected animated short films with positive or negative emotional valence (this is a preliminary step, to reproduce the previous literature and to confirm the selected stimulus were adequate); (2) the relationship between participants’ baseline score on an Emotional Intensity Scale and the potential change in their main emotion after watching animated short films with a positive or negative content; and (3) the association between their initial main emotion valence and the potential change in this emotion with participants’ sociodemographic information, as an exploratory analysis. To tackle these three goals, a randomized online experiment was conducted evaluating participants’ emotional state before and after watching animated short films with positive or negative emotional valence. It was hypothesized that the selected films would induce overall emotion changes according to their valences and that this emotional change would be influenced by the baseline emotion intensity, in a way that emotion change would be more related to the lower intensity of baseline emotions.

## Materials and methods

### Participants

Participants over 18 years old were recruited using digital networks and apps: WhatsApp, Facebook, and Instagram. This work was approved by our institutional ethics committee (CAAE: 37825120.3.0000.0071). Participants who completed all steps and questionnaires of the study were included in the sample.

The research was performed during the pandemic for about 2 weeks between October and November 2020 using the REDCap online platform. The REDCap platform provided the complete setting of packed of materials used in the study: the films with positive and negative contents, the relaxation audio, and the assessments used. A link for the experiment was shared on social media, such as WhatsApp and Instagram. The single link provided access to the REDCap survey, in which participants were instructed to go through the complete experiment. Each step of the experiment was presented on a different page, and all pages were presented in sequence, in a way that participants had to complete all the pages in a single participation (there was no option to continue later). They had to click at the end of each page to go to the next. Experiments conditions were presented one after the other. Only participants who went through all the films and questionnaires were included in the study.

### Experimental design

The short animated films were selected on youtube.com. The videos were selected for having similar duration, for mainly being related to different emotional valences, and for not having speech. They confirm if these films were adequate for the goals of this study, a pilot analysis was carried out among 13 researchers from the Neuroscience and Behavior Laboratory at Hospital Israelita Albert Einstein. Considering mainly the specific aims of this research, two videos with better emotional valence, positive or negative, were chosen. To evaluate the emotional modulation of short animated films, first, participants answered a questionnaire in order to evaluate their baseline emotional state (pre). Second, each individual was randomly assigned to groups that watched a short animated film with either positive or negative emotional valence, answering the same questionnaire right after the end of the film (post-film). The positive emotional content film was 2 min 28 s long (film: best inspiring animated short film volunteer your time), and the negative emotional content film was 2 min 43 s long (film: CGI animated short film: “Bruised” by Rok Won Hwang, Samantha Tu | CGMeetup). Third, participants received a relaxation intervention, and once again, they responded to the same questionnaire about their emotional state (post-relaxation).

Although the study has three phases, the post-relaxation phase was planned to reduce possible negative effects of the films (especially the negative ones). The relaxation was used to enable participants to leave the study in a good mood in case they were impacted by the negative film.

### Questionnaire

All participants provided information about their sociodemographic profile and about their emotional awareness (Supplementary material 1). Participants were questioned about their daily activities (working or studying) during social distancing, as well as about preexisting psychiatric disorders and daily medication intake.

An emotional state scale developed for this study, was applied. The participants were asked about the intensity of either different emotions, in a self-report, using a 10-point Likert scale (0 = not at all; 10 = extremely): happiness/joy,peacefulness/calm, satisfaction, pleasure/motivation, sadness, anger, fear, and disgust/aversion. Participants were instructed to base their evaluation on how they were feeling at the exact moment of answering the questionnaire. In addition, participants were asked which of these emotions was their prevailing one at that moment and what they thought was the cause of such feeling.

In order to assess participants’ emotional state, the emotional intensity scores were summed, in a way that the higher the score (max. 80 points), the more negative the emotional state (positive items were inverted before summing with negative items). The positive items were happiness/joy, peacefulness/calm, satisfaction, and pleasure/motivation. The negative items were sadness, anger, fear, and disgust/aversion.

The emotional awareness is an important score to know whether the individuals perceive and comprehend their emotions. This score was generated by summing the points attributed to the four sentences: I am aware of my emotions/I pay attention to how I feel when I have an emotion/When I feel an emotion, I can recognize it/When I feel tension, I pay attention to the changes that take place in my body (max. 40 points/each question max. 10 points), with a higher score indicating higher emotional awareness.

### Statistical analysis

Values of Cronbach’s alpha were calculated to evaluate the consistency of the questions regarding awareness of emotions and those assessing emotional state, using the data acquired before watching the animated short film. Then, to study the emotional state change after watching the films, the overall score from the emotional state scale was used as the dependent variable in a trimmed-means robust analysis of variance (ANOVA) (Mair and Wilcox, 2020), with the film group (positive and negative) and time (pre, post-film, and post-relaxation) as independent variables. This robust method is indicated for dealing with violations of normality and homoscedasticity (which were identified in the assumption check tests in section “Testing ANONA assumptions” of Supplementary material 2). As follow-up tests for the ANOVA, Wilcoxon rank-sum and signed-rank tests were used for pairwise comparisons, using the Bonferroni correction for multiple comparisons. To check whether following social distancing or working/studying during the pandemic could also affect the score from the emotional state scale, Kolmogorov–Smirnov tests were used in the pre-film data. In case of a significant effect, a proportion test would be used to check whether the variable was balanced across film groups.

Is the emotional state scale score at baseline different between participants who have or have not changed their emotional state after watching the film? To address this question, it was evaluated the relationship between the emotional score before watching the film and emotional change after the film with a two-way robust iterated re-weighted least squares (IWLS) ANOVA. Again, a robust method was chosen to deal with violations of normality assumption (section “Plots to check model assumptions” of Supplementary material 2).

The total score before the film was the dependent variable; this analysis had two independent variables, one indicating whether the volunteer had the same main emotion before and after the film (group Keep, for those who Keep; or group Change, for those who changed their emotions) and another indicating the valence of the main emotion before the film (positive or negative). The single-option questions about the prevailing emotion at each moment defined the Keep and Change groups, and even changes between emotions with the same valence were classified as Change. For example, we can have a change from anger to sadness even though both are in the negative valence. The main effects and their interaction were evaluated. We expect that the interaction is related to our second hypothesis in which participants who had high scores (intense negative emotion) and participants with lower scores (intense positive emotion) would be less likely to change their emotional state. The interaction would reflect that the emotional score depends on the combination of the emotional valence and whether participants may keep or change their emotions. For example, participants who keep their emotions would have higher scores if they had an initial negative valence or lower scores if they had an initial positive valence.

Furthermore, this analysis was repeated, with the addition of sociodemographic variables (gender, age, education level, marital status, use of medication, psychiatric disorder, currently working/studying, following social distancing recommendations, and the score of emotional awareness), to assure that they were not affecting the analysis.

As exploratory analysis to better describe and to identify possible sociodemographic associations with a potential emotional state change after the film, separate similar two-way models were calculated using each sociodemographic variable as the dependent variable. An analysis using the film group as the dependent variable was also performed. As in the previous analysis, the independent variables were the emotional variation (group Keep or group Change) from before to after watching the film, and the valence of the emotion before watching the film (positive or negative). As the sociodemographic variables were of different data types, appropriate two-way models were used for each variable type: trimmed-means robust ANOVA for continuous; ordinal logistic regression for ordinal; and multinomial logistic regression for nominal variables.

All analyses were carried out in the R software environment, with RStudio (RStudio Team, 2021) and the following packages: base (R Core Team, 2021), ggplot2 (Wickham, 2016), ggpubr (Kassambara, 2020), ggstance (Henry et al., 2020), grateful (Rodríguez-Sánchez and Hutchins, 2020), here (Müller, 2020), lemon (Edwards, 2020), lubridate (Grolemund and Wickham, 2011), MASS (Venables and Ripley, 2002), nnet (Venables and Ripley, 2002), plyr (Wickham, 2011), psych (Revelle, 2020), remotes (Hester et al., 2020), rstatix (Kassambara, 2021), scales (Wickham and Seidel, 2020), sjPlot (Lüdecke, 2021), table1 (Rich, 2020), tidyverse (Wickham et al., 2019), and WRS2 (Mair and Wilcox, 2020). *P*-values < 0.05 were considered as significant.

## Results

### Sample

**FIGURE 1:**
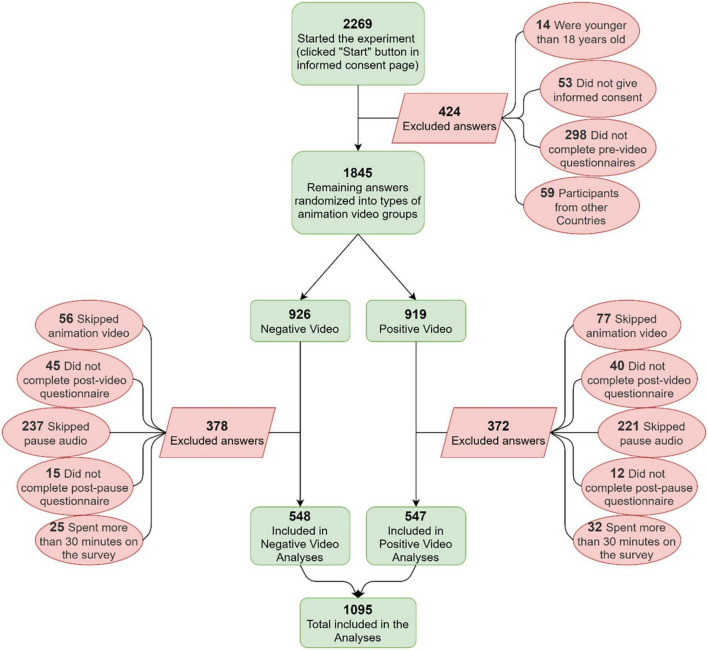
Study inclusion flowchart.

An initial sample of 2,269 participants who started the online experiment underwent the application of the exclusion criteria (Figure 1).

The final sample consisted of 1,095 Brazilian volunteers from 18 to 81 years old (mean age = 40.8 years). As presented in Table 1, most participants (85%) were women, were living in the state of São Paulo (Brazil, 75%), had monthly income above 3,135.00 Brazilian Reais (70%), were following social distancing recommendations (88%), and were either studying or working (85%). Regarding marital status, most participants were either married (46%) or single (37%). Diagnostic of psychiatric disorder was reported by almost a third of participants, and the most reported disorders were anxiety and depression. More than a fifth of the volunteers reported daily psychiatric medication intake (Table 1). Similar overall sociodemographic characteristics were observed in the excluded participants (Supplementary Table 1), although there might have been small differences, such as lower age groups (< 25 years), which represented 24% of the participants in the excluded sample and 17% in the analyzed sample.

**TABLE 1:** Sociodemographic characteristics.

	Total (*N* = 1,095)
Gender	
Female	926 (84.6%)
Male	161 (14.7%)
Other	3 (0.3%)
I would rather not answer	5 (0.5%)
Age (years)	
Mean (SD)	40.8 (14.0)
Median (Min, Max)	41.0 (18.0, 81.0)
Age groups (years)	
< 25	182 (16.6%)
25–34	175 (16.0%)
35–44	302 (27.6%)
45–54	230 (21.0%)
55–64	156 (14.2%)
≥ 65	50 (4.6%)
Education	
Lower secondary education (incomplete or complete)	5 (0.5%)
Higher secondary education (incomplete or complete)	124 (11.3%)
Undergraduate/bachelor (incomplete or complete)	404 (36.9%)
Graduate, MBA, M.Sc., Ph.D (incomplete or complete)	562 (51.3%)
Monthly income* (R$)	
0.00–1,045.00	56 (5.1%)
1,045.01–3,135.00	194 (17.7%)
3,135.01–6,270.00	227 (20.7%)
6,270.01–9,405.00	123 (11.2%)
9,405.01–12,540.00	105 (9.6%)
12,540.01–15,675.00	73 (6.7%)
15,675.00 or more	200 (18.3%)
I would rather not answer	117 (10.7%)
Marital status	
Married	500 (45.7%)
Divorced	163 (14.9%)
I would rather not answer	8 (0.7%)
Single	403 (36.8%)
Widow/Widower	21 (1.9%)
Is currently working or studying	
No	167 (15.3%)
Yes	928 (84.7%)
Is following social distancing recommendations	
No	128 (11.7%)
Yes	967 (88.3%)
Psychiatric disorders	
Anxiety	303 (27.7%)
Depression	171 (15.6%)
Bipolar disorder	15 (1.4%)
Other(s)	42 (3.8%)
None	714 (65.2%)
Uses psychiatric medication	
No	865 (79.0%)
Yes	230 (21.0%)

Max, maximum; min, minimum; SD, standard deviation.

*Brazilian minimum wage at the time of questionnaire application: R$1,045.00.

There were high values of Cronbach’s alpha, indicating consistency of the emotional awareness questions (raw alpha = 0.85) as well as of the emotion intensity questions (raw alpha = 0.88, section “Evaluation of consistency with Cronbanch’s alpha” of Supplementary material 2).

According to the Kolmogorov–Smirnov test, we did not find evidence for different score distributions between participants that reported following social distancing and those that did not (*p* = 0.494). Considering the participants that were working/studying during the pandemic, they scored differently than those that were not, before watching the films (working/studying = 28.1 ± 15.7; not working/studying = 31.8 ± 17.2; *p* = 0.0349). However, the proportion test indicated that the number of participants working/studying did not differ between film groups (positive film group = 83.7%; negative film group = 85.8%; *p* = 0.3934), suggesting this variable will not have a major impact in the comparison between groups.

### Emotional state changes after watching films with positive or negative emotional valence

**FIGURE 2:**
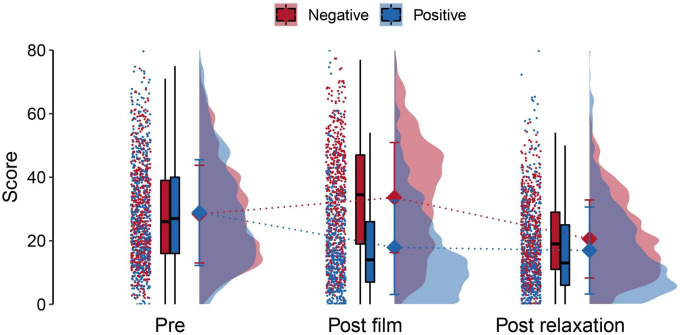
Total scores of the emotional state scale before the experiment (pre), after watching the animated short films (post-films), and after the relaxation (post-relaxation) in the positive (blue) and negative (red) short animated film groups. Dots to the left of each phase of the experiment represent scores of each participant at each experimental phase. Boxplots contain the median and first and third quantiles for each group at each experimental phase. Diamonds represent the mean scores of each group at each experimental phase, and error bars represent the standard deviation of the mean, and these are accompanied by kernel density estimates of the data distribution. The ANOVA revealed a significant main effect of the film group (*p* < 0.001), a main effect of time (*p* < 0.001), and a significant film group by time interaction (*p* < 0.001).

The trimmed-means robust ANOVA used to analyze the film and the relaxation effects on the emotional state revealed significant main effects of the film group, time, and a significant interaction effect [*p* < 0.001, Supplementary material 2 (Supplementary Table 4)]. We found no difference between groups before watching the films in the follow-up tests (mean ± standard deviation: negative = 28.389 ± 15.377; positive = 28.874 ± 16.647; corrected *p* = 1). After watching the film, the negative group presented higher emotional state scores than the positive group (negative = 33.604 ± 17.325; positive = 17.916 ± 14.824; corrected *p* < 0.001), and this difference was Keep post-relaxation (negative = 20.577 ± 12.286; positive = 16.945 ± 13.696; corrected *p* < 0.001), although the difference decreased post-relaxation (Figure 2). In each group, all differences were significant when comparing times (pre, post-film, and post-relaxation) (positive group post-film vs. post-relaxation corrected *p* = 0.002; all other corrected *p* < 0.001). The effect of the relaxation was smaller in the positive group, and the film effect seemed smaller in the negative group (Supplementary material 2).

### Association between participants’ baseline score on the emotional state scale and the possible change in their main emotion after watching films with a positive or negative content

The two-way robust iterated re-weighted least squares (IWLS) ANOVA used to estimate the pre-emotional score relation with the emotional change after the film showed significant effects of the emotional valence (*p* < 0.001) as well as of its interaction with emotional variation (*p* < 0.001), but the effect of emotional variation by itself did not reach significance [*p* = 0.071, Supplementary material 2 (Supplementary Table 11)]. The *post hoc* tests showed higher emotional state scale scores for participants with an initial negative compared with positive main emotion for both Keep and Change groups (Keep: negative valence = 45.224 ± 13.023; positive valence = 17.941 ± 9.449; Change: negative valence = 41.107 ± 12.297; positive valence = 18.993 ± 9.603; corrected *p*-values < 0.001). In addition, for participants who had a negative main emotion before watching the film, the Keep group had a higher emotional state scale score at baseline than the Change group (corrected *p* = 0.012). This effect was not significant for participants who had a positive main emotion (corrected *p* = 0.196). Similar effects of emotional valence and of the intervention were observed when including the sociodemographic variables in the model (Supplementary material 2).

### Association between the initial main emotion valence and the potential change in this emotion with participants’ sociodemographic information

Table 2 presents clinical and sociodemographic variables for both Keep and Change groups and also according to their baseline main emotion (positive or negative). The Keep group comprises 31% of the sample.

**TABLE 2:** Sociodemographic analysis.

Initial emotional state	Group keep		Group change	
	Negative	Positive	Negative	Positive
	(*n* = 134)	(*n* = 204)	(*n* = 328)	(*n* = 429)
Age*^,1,2,3^				
Mean (SD)	38.3 (13.1)	45.2 (13.2)	38.2 (14.5)	41.4 (13.7)
Median (Min, Max)	38.0 (18.0, 72.0)	46.0 (18.0, 74.0)	38.0 (18.0, 76.0)	41.0 (18.0, 81.0)
Gender**^1^				
Female	111 (82.8%)	163 (79.9%)	284 (86.6%)	368 (85.8%)
Male	23 (17.2%)	39 (19.1%)	44 (13.4%)	55 (12.8%)
Other	0 (0%)	1 (0.5%)	0 (0%)	2 (0.5%)
I would rather not answer	0 (0%)	1 (0.5%)	0 (0%)	4 (0.9%)
Marital status^†,2^				
Married	49 (36.6%)	100 (49.0%)	136 (41.5%)	215 (50.1%)
Divorced	14 (10.4%)	37 (18.1%)	49 (14.9%)	63 (14.7%)
I would rather not answer	0 (0%)	1 (0.5%)	4 (1.2%)	3 (0.7%)
Single	67 (50.0%)	61 (29.9%)	131 (39.9%)	144 (33.6%)
Widow/Widower	4 (3.0%)	5 (2.5%)	8 (2.4%)	4 (0.9%)
Level of education^‡,2^				
Lower secondary education	1 (0.7%)	1 (0.5%)	0 (0%)	3 (0.7%)
Higher secondary education	20 (14.9%)	17 (8.3%)	45 (13.7%)	42 (9.8%)
Undergraduate/Bachelor	61 (45.5%)	69 (33.8%)	127 (38.7%)	147 (34.3%)
Graduate, MBA, M.Sc., Ph.D	52 (38.8%)	117 (57.4%)	156 (47.6%)	237 (55.2%)
Is following social distancing recommendations**^,1^				
No	16 (11.9%)	36 (17.6%)	32 (9.8%)	44 (10.3%)
Yes	118 (88.1%)	168 (82.4%)	296 (90.2%)	385 (89.7%)
Is currently working or studying**				
No	25 (18.7%)	30 (14.7%)	57 (17.4%)	55 (12.8%)
Yes	109 (81.3%)	174 (85.3%)	271 (82.6%)	374 (87.2%)
Psychiatric disorders**^,2^				
No	77 (57.5%)	160 (78.4%)	191 (58.2%)	306 (71.3%)
Yes	57 (42.5%)	44 (21.6%)	137 (41.8%)	123 (28.7%)
Uses psychiatric medication**^,2,3^				
No	94 (70.1%)	179 (87.7%)	250 (76.2%)	342 (79.7%)
Yes	40 (29.9%)	25 (12.3%)	78 (23.8%)	87 (20.3%)
Emotional awareness*^,2^				
Mean (SD)	29.0 (8.92)	32.4 (5.58)	29.9 (7.10)	31.4 (5.80)
Median (Min, Max)	31.0 (0, 40.0)	33.0 (11.0, 40.0)	31.0 (1.00, 40.0)	32.0 (0, 40.0)
Group**^,1,3^				
Negative film	83 (61.9%)	100 (49.0%)	142 (43.3%)	223 (52.0%)
Positive film	51 (38.1%)	104 (51.0%)	186 (56.7%)	206 (48.0%)

Max, maximum; min, minimum; SD, standard deviation.

*Trimmed-means robust ANOVA.

**Logistic regression.

^†^Multinomial logistic regression.

^‡^Ordinal logistic regression.

^1^Significant effect of emotional variation.

^2^Significant effect of emotional valence.

^3^Significant interaction between variation and valence of emotion.

Table 2 also presents the results of the statistical analyses, which evaluated the association of each sociodemographic variable with potential emotional variation (Keep and Change groups); the valence of the main emotion of the individual at baseline (negative or positive); and the interaction between variation and valence. Detailed results of the analysis model for each sociodemographic variable are presented in Supplementary material (section “The association between the initial main emotion valence and their potential change in this emotion with participants’ sociodemographic information” of Supplementary material 2).

Significant variation effects were found for age, gender, social distancing, and film group [Supplementary material 2 (Supplementary Table 27)]. Keep group participants were older than those from the Change group (Keep: 42.5 ± 13.5; Change: 40.0 ± 14.2). In addition, the Keep group had more participants from the negative film group (Keep: 54.1%; Change: 48.2%), had more men (Keep: 18.3%; Change: 13.1%), and had a lower percentage of participants following social distancing recommendations than the Change group (Keep: 84.6%; Change: 90.0%). A valence effect was observed for age, marital status, level of education, psychiatric disorders, use of psychiatric medication, and emotional awareness score [Supplementary material 2 (Supplementary Table 28)]. Participants with positive pre-film main emotion were on average older (positive: 42.7 ± 13.7; negative: 38.2 ± 14.1) and had stronger emotional awareness than the negative emotion group (positive: 31.7 ± 5.7; negative: 29.7 ± 7.7). Also, there were a higher number of married participants in the positive main emotion group compared with the negative main emotion group (positive: 49.8%; negative: 40.0%). The negative emotion group had a lower level of education than the positive emotion group and reported more frequently to have a psychiatric disorder (negative: 44.1%; positive: 30.0%) and be using psychiatric medication (negative: 25.5%; positive: 17.7%) (Table 2).

The interaction effect was significant for age, psychiatric medication intake, and film group. The age difference between positive and negative emotion participants was higher in the Keep group, as whose participants with positive valence were older on average. Also, positive Keep group participants seemed to use less psychiatric medication, while negative Change group participants were those more likely to use psychiatric medication (Table 2).

In addition, although most of the participants had changed their initial emotion (69.1%), of those who were in a negative valance emotion and changed their initial emotion, a higher proportion (56.7%) watched a positive valence film. Of those who were in a negative emotion and kept this initial emotion, a higher proportion (61.9%) watched a negative valence film. Among the participants who presented an initial positive emotion and kept it, the proportion of participants watching either positive or negative valence films was similar. A similar pattern was an observer of participants with an initial positive emotion in the Change group (Table 2).

## Discussion

This study evaluated how animated short films with positive and negative contents affect the emotional state during the COVID-19 pandemic. The study evaluated (1) how participants’ emotional state changes after watching animated short films with positive or negative emotional valence; (2) the relationship between participants’ baseline score on an Emotional Intensity Scale and the potential change in their main emotion after watching animated short films with a positive or negative content; and (3) the association between the initial main emotion valence and the potential change in this emotion with participants’ sociodemographic information. To address these issues, we measured the emotional state of the participants in three moments: before watching the films with a positive or negative content, after watching them, and after practicing a relaxation. Overall, we found that animated short films were able to change participants’ emotional state in the direction of the valence of the film: positive films lead to an improved and negative films lead to a worsened emotional state. In addition, we found that participants that reported to be with a main negative emotion and did not change this emotion after watching the film had a more intense negative emotion than those who did change their main emotion after watching the film.

Watching a film with negative valence induces participants to be in a more negative emotional state than at baseline. These results are in line with previous studies of our research group using only audios reporting news with positive and negative contents (Bazán et al., 2021a,b). There is a variety of ways to elicit emotion; for example, Westermann et al. (1996) carried out many procedures using films, imagination strategies, storytelling, music, feedback, social interaction, posed facial expressions, and others. In this study, the film was the chosen method for eliciting emotions. It was taken into consideration in this study that watching audiovisual stimuli evokes real emotions; thus, it generates more ecological stimuli (Negrão et al., 2021). In psychology, an ecological stimulus is a measure of how test performance predicts behaviors in real-world settings. It is relevant to mention that the films used in the present study had audio sound; however; they were speechless, which suggests that the effects of the valence of the stimuli are not restricted to the audio speech form. Other studies on emotions have used different ways to elicit emotional responses in the laboratory: featured films (Gross, 2008), others with animated social media (Stark, 2018), or Graphical Interchange Format (GIF) (Yang et al., 2022).

In our study, some participants maintain their emotions even after watching the films (Keep group). Participants keep the same negative emotion from the beginning of the experiment were the ones with a higher (and thus more negative) score on the emotional state scale. These results are in line with previous studies showing that emotional control strategies are less successful when there is an intense negative emotion (Shafir et al., 2015). In addition, individuals under depression and anxiety tend to have an attention bias toward information with negative valence (Gasper and Clore, 2002; Paulus, 2007; Paulus and Angela, 2012). Finally, individuals under depression have shown a decreased processing of valuable positive information (Piazzagalli et al., 2008; Disner et al., 2011).

Negative events may trigger a maladaptive response such as rumination, which is immersing in a repetitive thought that leads to interpreting the event in a more negative way. It has been shown that rumination is a risk factor for psychopathology (Cohen et al., 2014). This rumination process can be in line with the theoretical construct of the refractory period for our emotional experience (Ekman and Cordaro, 2011). According to this construct, individuals under a stronger emotion could enter the refractory period, a state in which people are less affected by events that could modulate their current emotional state. The refractory period filters the information available, letting in only the information for which the emotional valence is compatible with the one being experienced (Kuppens et al., 2010). Therefore, a person in a refractory period is expected to be less prone to change their emotional state after receiving a stimulus with an opposite emotional value. Scientific evidence of this condition may be useful for a better understanding of aspects of social cognition and its implications in human interaction. In the scientific field, a practical implication may be the bias caused by this effect of emotion in an evaluation of psychological tests and should be controlled.

Complementary research on emotion inertia, that is, a stronger autoregressive slope of emotions, thus with lower wellbeing, indicated that higher emotion inertia for negative emotion stimuli means a longer period of emotion over time, but higher emotion inertia for positive emotions does not predict lower wellbeing as measured by depressive symptoms and life satisfaction. High emotional inertia, much like being stuck in a refractory period, makes it hard to switch the emotional experience even when the context has changed (Koval et al., 2016).

Considering the valence of participants’ main emotion at baseline, the present study showed that participants had higher Emotional Intensity Scale scores when under a negative emotion, but lower scores when under a positive emotion. This was expected, as the scale was built to present negative emotions as high scores and positive emotions as low scores. This result indicates that the scale is consistent with the single main emotion question. Our Emotional Intensity Scale scores were not validated in an independent study; however, we had an internal consistency of 0.88.

We also investigated the association between the initial main emotion valence and the potential change in this emotion (variation) with participants’ sociodemographic information. The interaction between valence and variation was significant for age and use of psychiatric medication, with the positive Keep group being older and having less participants who declared psychiatric medication intake. In accordance with the maturity perspective, people usually present a change in emotions from negative to positive as well as from active to passive or more serene, and this study reflects this expectation about participants’ emotions (Ross and Mirowsky, 2008). In addition, elders present higher mood stability, emotional control, emotional maturity through moderation, positive effect, lower responsiveness, and sensation seeking (Lawton et al., 1992). These findings are consistent with the hypothesized increase in self-regulatory capacity with age.

In general, positive valence was related to older age and to less declared use of psychiatric medication. Similarly, positive valence groups were associated with higher education levels, higher emotional awareness, being married, and less psychiatric disorders. Married people are usually healthier and live longer than those who live alone, including better mental health, less health problems, and faster recovery from diseases, especially in happy marriages (Dupre et al., 2009; Lawrence et al., 2019). Due to the COVID-19 pandemic, many communities-imposed quarantine measures to mitigate the spread of the coronavirus, leading to social isolation. In a study with 1,013 USA adults, they filled in UCLA Loneliness Scale-3 and Public Health Questionnaire (PHQ-9). Loneliness was elevated (43%) and was strongly associated with greater depression and suicidal ideation. Loneliness is a critical public health concern during a pandemic (Killgore et al., 2020).

The relaxation audio had a positive effect as both groups presented smaller scores after the relaxation, even when compared with the pre-film scores. Although this is a secondary result in the current study, it is important to highlight that in the current design, participants finished the experiment in a more positive state than when they started. This should be a concern when using stimulus which can induce negative emotions during the experiment. The main goal of including the relaxation audio was to reduce the possible negative effects of watching the negative valence film.

The preset study has some limitations. There was approximately 50% loss of the initial sample; it was probably due to the fact of being an online experiment, in which several aspects could have caused participants to start, but not complete the experiment. In a study about abandon rates in online surveys, they increase for that took more than 7–8 min to complete (Chudoba, 2022). This could be related to internet connection issues (broken connection during the experiment). Furthermore, some of the participants might have opened the link without having enough time to finish the experiment (out of curiosity), and some of the participants might have been interrupted during the experiment. Participants were mainly married Brazilian women aged between 35 and 40 years with the higher educational level (graduation/MBA/M.Sc.,/Ph.D). Although there could have been a bias due to attrition, the excluded participants presented similar sociodemographic characteristics. This study might have had different results if it had assessed a population with different sociodemographic characteristics. The negative film did not have as much effect as the positive film, given that more participants in the Keep group watched the negative film, and almost half of the positive Keep group watched a negative film compared with less than 40% of participants who kept a negative emotion after watching the positive film. Indeed, the negative film may not have a strong explicit content, and consequently, they might not have affected all the participants of this study to get them out of this basal emotional state. Furthermore, the participants’ perception of the film was not directly assessed, and participants could have interpreted or perceived the film in ways different from the expected positive or negative valences. It is also expected that the animation films engage more than one emotion. However, even with this difference, the groups were randomized in similar ways, minimizing the variation bias. Furthermore, this study did not use neutral films that did not contain emotional valence. Due to the lack of this control condition, it was not possible to measure to what extent the emotional state would have changed with a neutral valence movie.

## Conclusion

With the aim of investigating the emotional state changes after watching animated short films with positive or negative emotional valence during the COVID-19 pandemic, the study results suggest that it is important to be aware of the emotional valence of animated short films, which may directly affect the emotional state.

After watching a film with negative valence, participants were in a more negative emotional state than at baseline and compared with those who watched the film with positive valence. These findings suggest that watching a film with negative valance can negatively affect the emotional state. Also, individuals who had a negative baseline emotion and maintained the same emotion after the film presented higher baseline emotional state scores (more negative state) than those who changed their emotions. It may show that individuals who kept the emotion may be associated with an initially stronger emotional state.

## Data availability statement

The original contributions presented in this study are included in the article/Supplementary material, further inquiries can be directed to the corresponding author.

## Ethics statement

The studies involving human participants were reviewed and approved by the Ethics and Research Committee of Albert Einstein Hospital. The patients/participants provided their written informed consent to participate in this study.

## Author contributions

EK, JN, PB, EE, and RA formulated the research questions, designed the study, and contributed and agreed to the final manuscript. PB and RA curated the data and analyzed the data and wrote the first draft. JN wrote the first draft. EK supervised the project. All authors revised the manuscript and approved the submitted version.
