# Supplementary material for: Baseline emotional state influences on the response to animated short films: A randomized online experiment
Source: Front Psychol. 2022 Dec 15;13:1009429. doi: 10.3389/fpsyg.2022.1009429 (PMC9797995; doi:10.3389/fpsyg.2022.1009429)
Supplement: Supplementary file 1 [file Data_Sheet_1.DOCX]

**Questionnaire 1 – Social demographic data**

1. Age: __________
2. Gender:

( ) Female

( ) Male

( ) Rather not to tell

( ) Other

1. Marital state:

( ) Single

( ) Married / Stable union (cohabitation / civil partnership)

( ) Divorced / Separated

( ) Widowed

( ) Rather not to tell

1. Ethnicity

( ) White

( ) Brown

( ) Black

( ) Yellow

( ) Indigenous

( ) Rather not to tell

( ) Other __________________________

1. Country of residency

_________________________________

1. State of residency

_________________________________

1. Education:

( ) Elementary School (incomplete or complete)

( ) Middle School (incomplete or complete)

( ) High School (incomplete or complete)

( ) University (incomplete or complete)

( ) Post graduation (incomplete or complete)

1. Family income:

( ) R$ 0,00 - R$ 1045,00

( ) R$ 1045,01 - R$ 3135,00

( ) R$ 3135,01 – R$ 6270,00

( ) R$ 6270,01 – R$ 9405,00

( ) R$ 9405,01 – R$ 12540,00

( ) R$ 12540,01 – R$ 15675,00

( ) R$ 15675,01 – or more

( ) Rather not to tell

1. Do I have a psychiatric diagnosis?

( ) yes ( ) no

( ) Depression

( ) Anxiety

( ) Bipolar Disorder

( ) Schizophrenia

( ) Other. Which one? ________________________________

1. Do I take psychiatric medication?

( ) yes ( ) no

1. Am I or have been in social isolation or social distancing?

( ) yes ( ) no

1. Am I working or studying in this period of the pandemics?

( ) yes ( ) no

**Questionnaire 2 –Emotional State**

To answer the following questions, notice how you are you feeling right now.

1. I’m aware of my emotions

0 1 2 3 4 5 6 7 8 9 10

Not More or less Extremely

even a little

1. I pay attention to how I feel when I have an emotion

0 1 2 3 4 5 6 7 8 9 10

Not More or less Extremely

even a little

1. When I feel an emotion, I can recognize it.

0 1 2 3 4 5 6 7 8 9 10

Not More or less Extremely

even a little

1. When I feel tension, I pay attention to the changes that take place in my body

0 1 2 3 4 5 6 7 8 9 10

Not More or less Extremely

even a little

1. How intensely are you feeling these emotions:

Happiness / Joy / Enthusiastic

0 1 2 3 4 5 6 7 8 9 10

Not at all More or less Very Intense

Peace / Tranquility / Calm

0 1 2 3 4 5 6 7 8 9 10

Not at all More or less Very Intense

Contentment / Satisfaction / Plenitude

0 1 2 3 4 5 6 7 8 9 10

Not at all More or less Very Intense

Pleasure / Amusement / Motivation

0 1 2 3 4 5 6 7 8 9 10

Not at all More or less Very Intense

Sadness / Discouragement/ Loneliness

0 1 2 3 4 5 6 7 8 9 10

Not at all More or less Very Intense

Anger / Irritation / Frustration

0 1 2 3 4 5 6 7 8 9 10

Not at all More or less Very Intense

Fear / Anxiety / Panic

0 1 2 3 4 5 6 7 8 9 10

Not at all More or less Very Intense

Disgust / Aversion / Contempt

0 1 2 3 4 5 6 7 8 9 10

Not at all More or less Very Intense

1. Which emotions are influencing you the most at the moment?

( ) Happiness / Joy / Enthusiastic

( ) Contentment / Satisfaction / Plenitude

( ) Pleasure / Amusement / Motivation

( ) Sadness / Discouragement/ Loneliness

( ) Anger / Irritation / Frustration

( ) Fear / Anxiety / Panic

( ) Disgust / Aversion / Contempt

1. What caused me this?

( ) Work stress

( ) Pandemic

( ) Bad news

( ) My boyfriend/girlfriend/ husband/ wife

( ) My children

( ) Other __________________________

1. How intensely can I notice body changes when I feel these emotions that influence me right now (eg: changes in breathing, tension or relaxation, temperature, etc)

0 1 2 3 4 5 6 7 8 9 10

Not More or less Extremely

even a little

1. Now you will watch a film of approximately 2 minutes long
2. In the following questions, answer now, at this moment:

Questionnaire 3 – Emotional Intensity Scale

Had I watched this specific film previously?

Yes ( ) No ( )

1. How intensely are you feeling these emotions:

Happiness / Joy / Enthusiastic

0 1 2 3 4 5 6 7 8 9 10

Not at all More or less Very Intense

Peace / Tranquility / Calm

0 1 2 3 4 5 6 7 8 9 10

Not at all More or less Very Intense

Contentment / Satisfaction / Plenitude

0 1 2 3 4 5 6 7 8 9 10

Not at all More or less Very Intense

Pleasure / Amusement / Motivation

0 1 2 3 4 5 6 7 8 9 10

Not at all More or less Very Intense

Sadness / Discouragement/ Loneliness

0 1 2 3 4 5 6 7 8 9 10

Not at all More or less Very Intense

Anger / Irritation / Frustration

0 1 2 3 4 5 6 7 8 9 10

Not at all More or less Very Intense

Fear / Anxiety / Panic

0 1 2 3 4 5 6 7 8 9 10

Not at all More or less Very Intense

Disgust / Aversion / Contempt

0 1 2 3 4 5 6 7 8 9 10

Not at all More or less Very Intense

1. Which emotions are influencing you at the moment?

( ) Happiness / Joy / Enthusiastic

( ) Contentment / Satisfaction / Plenitude

( ) Pleasure / Amusement / Motivation

( ) Sadness / Discouragement/ Loneliness

( ) Anger / Irritation / Frustration

( ) Fear / Anxiety / Panic

( ) Disgust / Aversion / Contempt

1. What caused me this?

( ) Work stress

( ) Pandemic

( ) Bad news

( ) My boyfriend/girlfriend/ husband/ wife

( ) My children

( ) Other __________________________

1. How intensely can I notice bodily changes when I feel these emotions that influence me right now (eg: changes in breathing, tension or relaxation, temperature, etc)

0 1 2 3 4 5 6 7 8 9 10

Not More or less Extremely

even a little

1. Now, you will listen to an audio relaxation focusing on you breathing. It can be used for breaks during daily routine. Try to follow the audio instructions

**Questionnaire 3 – Emotional Intensity Scale**

Had I heard this specific audio previously?

Yes ( ) No ( )

In the following questions, answer how you feel right now:

1. How intensely are you feeling these emotions:

Happiness / Joy / Enthusiastic

0 1 2 3 4 5 6 7 8 9 10

Not at all More or less Very Intense

Peace / Tranquility / Calm

0 1 2 3 4 5 6 7 8 9 10

Not at all More or less Very Intense

Contentment / Satisfaction / Plenitude

0 1 2 3 4 5 6 7 8 9 10

Not at all More or less Very Intense

Pleasure / Amusement / Motivation

0 1 2 3 4 5 6 7 8 9 10

Not at all More or less Very Intense

Sadness / Discouragement/ Loneliness

0 1 2 3 4 5 6 7 8 9 10

Not at all More or less Very Intense

Anger / Irritation / Frustration

0 1 2 3 4 5 6 7 8 9 10

Not at all More or less Very Intense

Fear / Anxiety / Panic

0 1 2 3 4 5 6 7 8 9 10

Not at all More or less Very Intense

Disgust / Aversion / Contempt

0 1 2 3 4 5 6 7 8 9 10

Not at all More or less Very Intense

1. Which emotions are influencing you at the moment?

( ) Happiness / Joy / Enthusiastic

( ) Contentment / Satisfaction / Plenitude

( ) Pleasure / Amusement / Motivation

( ) Sadness / Discouragement/ Loneliness

( ) Anger / Irritation / Frustration

( ) Fear / Anxiety / Panic

( ) Disgust / Aversion / Contempt

1. What caused me this?

( ) Work stress

( ) Pandemic

( ) Bad news

( ) My boyfriend/girlfriend/ husband/ wife

( ) My children

( ) Other __________________________

1. How intensely can I notice bodily changes when I feel these emotions that influence me right now (eg: changes in breathing, tension or relaxation, temperature, etc)

0 1 2 3 4 5 6 7 8 9 10

Not More or less Extremely

even a little
