# Supplementary material for: Baseline emotional state influences on the response to animated short films: A randomized online experiment
Source: Front Psychol. 2022 Dec 15;13:1009429. doi: 10.3389/fpsyg.2022.1009429 (PMC9797995; doi:10.3389/fpsyg.2022.1009429)
Supplement: Supplementary file 2 [file Data_Sheet_2.PDF]

Emotional state in response to animated short films - Supplementary Material 1

Juliana Negrão, Paulo Rodrigo Bazán, Raymundo Machado de Azevedo Neto, Shirley Silva Lacerda, Elisa Harumi Kozasa

04/01/2022 (mm/dd/yyyy)

In this supplementary report, we show the detailed results from the online experiment where participants responded a questionnaire at three time points: before watching a short animation film, after watching a short animation film, and after undergoing a relaxation audio intervention. There were two short animation films with different emotional valences: one with a more positive emotional content; and one with a more negative emotional content. Participants were randomly assigned to groups that watched positive or negative short animation films.

Our main measurements of interest were the scores to a questionnaire developed by our group to assess the emotional state intensity (Supplementary Material 2). The questionnaire contained itens scored on a Likert-scale from 0 to 10, asking participants to report the intensity of the following emotions: Happiness/Joy, Peacefulness/Calm, Satisfaction, Pleasure/Motivation, Sadness, Anger, Fear and Disgust/Aversion. An overall score was calculated for each participant by summing their responses to all items (positive items were inverted before summing with negative items). The positive items were Happiness/Joy, Peacefulness/Calm, Satisfaction and Pleasure/Motivation. The negative items were Sadness, Anger, Fear and Disgust/Aversion

1. Evaluation of consistency with Cronbach’s alpha

This section presents the detailed outputs from calls to alpha function in psych package (https://cran.r-project.org/package=psych), which calculates the Cronbach’s alpha of the questionnaires, including an evaluation of reliability if an item is dropped.

1.1. Awareness of emotions

Score based on 4 questions about awareness of emotions (Supplementary Material 2).

```
##
## Reliability analysis
## Call: alpha(x = Emotion_data_clean[31:34], check.keys = TRUE)
##
##   raw_alpha std.alpha G6(smc) average_r S/N    ase mean  sd median_r
##       0.85      0.86    0.83       0.6 6.1 0.0072  7.7 1.7     0.58
##
##  lower alpha upper     95% confidence boundaries
## 0.84 0.85 0.87
##
##  Reliability if an item is dropped:
##          raw_alpha std.alpha G6(smc) average_r S/N alpha se   var.r med.r
## atencao1      0.81      0.81    0.75      0.59 4.3   0.0099 1.4e-03  0.59
## atencao2      0.78      0.79    0.71      0.55 3.7   0.0113 5.5e-05  0.55
## atencao3      0.83      0.84    0.79      0.64 5.3   0.0089 1.0e-02  0.63
## atencao4      0.84      0.84    0.79      0.63 5.1   0.0088 9.7e-03  0.59
##
##  Item statistics
##             n raw.r std.r r.cor r.drop mean  sd
## atencao1 1095  0.83  0.85  0.79   0.72  8.0 1.8
## atencao2 1095  0.88  0.88  0.85   0.78  7.8 1.9
## atencao3 1095  0.81  0.81  0.70   0.65  7.6 2.0
## atencao4 1095  0.83  0.81  0.71   0.66  7.4 2.3
```

Cronbach's alpha was high ($\alpha = 0.854$), suggesting that the sum of the items can be used to evaluate emotional awareness.

1.2. Emotions intensity (including all emotions)

Score based on 8 questions Likert-scale questions about the intensity of emotions experienced (Supplementary Material 2). Four questions regarded positive emotions, and four regarded negative emotions. The questions were answered 3 times: Before watching a short animation film (Pre); after watching the film (Post film); and after a relaxation audio (Post relaxation).

1.2.1 Pre

```
##
## Reliability analysis
## Call: alpha(x = Emotion_data_clean[35:42], check.keys = TRUE)
##
##   raw_alpha std.alpha G6(smc) average_r S/N    ase mean sd median_r
##       0.88      0.89    0.91      0.49 7.7 0.0054  3.6  2     0.48
##
##  lower alpha upper     95% confidence boundaries
## 0.87 0.88 0.89
##
##  Reliability if an item is dropped:
##              raw_alpha std.alpha G6(smc) average_r S/N alpha se var.r med.r
## felicidade        0.87      0.87    0.89      0.49 6.6   0.0060 0.028  0.49
## paz               0.86      0.86    0.89      0.47 6.3   0.0064 0.038  0.48
## contentamento     0.86      0.86    0.89      0.47 6.1   0.0064 0.030  0.48
## prazer            0.87      0.87    0.89      0.49 6.7   0.0060 0.026  0.49
## tristeza          0.86      0.86    0.89      0.48 6.3   0.0066 0.043  0.45
## raiva             0.86      0.87    0.89      0.48 6.5   0.0063 0.041  0.48
## medo              0.88      0.88    0.91      0.52 7.5   0.0055 0.038  0.53
## nojo              0.89      0.89    0.92      0.54 8.3   0.0054 0.029  0.54
##
##  Item statistics
##                  n raw.r std.r r.cor r.drop mean  sd
## felicidade    1095  0.74  0.76  0.75   0.66  4.2 2.4
## paz           1095  0.80  0.81  0.79   0.74  3.7 2.4
## contentamento 1095  0.82  0.83  0.83   0.75  4.4 2.6
## prazer        1095  0.74  0.76  0.75   0.65  4.7 2.5
## tristeza      1095  0.82  0.80  0.77   0.74  3.9 3.1
## raiva         1095  0.79  0.77  0.74   0.70  3.2 3.0
## medo          1095  0.68  0.66  0.59   0.55  3.2 3.0
## nojo          1095  0.56  0.56  0.47   0.45  1.3 2.3
```

1.2.2. Post film

```
##
## Reliability analysis
## Call: alpha(x = Emotion_data_clean[55:62], check.keys = TRUE)
##
##   raw_alpha std.alpha G6(smc) average_r S/N    ase mean  sd median_r
##       0.91      0.91    0.93      0.55 9.9 0.0041  3.2 2.2     0.52
##
##  lower alpha upper     95% confidence boundaries
## 0.9 0.91 0.92
##
##  Reliability if an item is dropped:
##                        raw_alpha std.alpha G6(smc) average_r  S/N alpha se
## felicidade_pos_video        0.89      0.89    0.91      0.54  8.1   0.0051
## paz_pos_video               0.89      0.89    0.92      0.53  8.0   0.0051
## contentamento_pos_video     0.89      0.89    0.91      0.53  7.9   0.0052
## prazer_pos_video            0.89      0.89    0.91      0.54  8.1   0.0050
## tristeza_pos_video          0.90      0.90    0.93      0.55  8.6   0.0047
## raiva_pos_video             0.90      0.90    0.91      0.56  8.7   0.0045
## medo_pos_video              0.91      0.91    0.94      0.60 10.4   0.0040
## nojo_pos_video              0.91      0.91    0.93      0.59 10.2   0.0041
##                        var.r med.r
## felicidade_pos_video    0.026  0.51
## paz_pos_video           0.033  0.49
## contentamento_pos_video 0.026  0.51
## prazer_pos_video        0.027  0.51
## tristeza_pos_video      0.045  0.47
## raiva_pos_video         0.043  0.51
## medo_pos_video          0.035  0.57
## nojo_pos_video          0.035  0.57
##
##  Item statistics
##                           n raw.r std.r r.cor r.drop mean  sd
## felicidade_pos_video    1095  0.86  0.84  0.85   0.80  4.2 2.9
## paz_pos_video           1095  0.85  0.85  0.84   0.80  3.7 2.8
## contentamento_pos_video 1095  0.88  0.87  0.87   0.83  4.1 3.0
## prazer_pos_video        1095  0.85  0.84  0.84   0.79  4.6 3.2
## tristeza_pos_video      1095  0.79  0.79  0.74   0.71  3.5 3.1
## raiva_pos_video         1095  0.76  0.78  0.75   0.69  2.3 2.8
## medo_pos_video          1095  0.62  0.64  0.56   0.52  2.1 2.6
## nojo_pos_video          1095  0.63  0.65  0.59   0.53  1.3 2.4
```

1.2.3. Post relaxation

```
##
## Reliability analysis
## Call: alpha(x = Emotion_data_clean[73:80], check.keys = TRUE)
##
##   raw_alpha std.alpha G6(smc) average_r S/N    ase mean  sd median_r
##       0.88      0.88    0.91      0.48 7.3 0.0054  2.3 1.6     0.46
##
##  lower alpha upper     95% confidence boundaries
## 0.87 0.88 0.89
##
##  Reliability if an item is dropped:
##                         raw_alpha std.alpha G6(smc) average_r S/N alpha se
## felicidade_pos_pausa         0.86      0.86    0.90      0.48 6.4   0.0064
## paz_pos_pausa                0.85      0.85    0.90      0.46 5.8   0.0067
## contentamento_pos_pausa      0.85      0.85    0.89      0.45 5.8   0.0070
## prazer_pos_pausa             0.86      0.86    0.89      0.48 6.3   0.0064
## tristeza_pos_pausa           0.86      0.86    0.90      0.47 6.2   0.0060
## raiva_pos_pausa              0.87      0.86    0.89      0.47 6.3   0.0057
## medo_pos_pausa               0.87      0.87    0.91      0.49 6.7   0.0056
## nojo_pos_pausa               0.88      0.88    0.91      0.52 7.6   0.0055
##                         var.r med.r
## felicidade_pos_pausa    0.033  0.46
## paz_pos_pausa           0.046  0.40
## contentamento_pos_pausa 0.036  0.46
## prazer_pos_pausa        0.032  0.46
## tristeza_pos_pausa      0.052  0.43
## raiva_pos_pausa         0.047  0.46
## medo_pos_pausa          0.048  0.46
## nojo_pos_pausa          0.037  0.47
##
##  Item statistics
##                            n raw.r std.r r.cor r.drop mean  sd
## felicidade_pos_pausa    1095  0.78  0.73  0.72   0.68 3.88 2.5
## paz_pos_pausa           1095  0.83  0.81  0.79   0.76 2.23 2.1
## contentamento_pos_pausa 1095  0.86  0.82  0.82   0.79 3.25 2.6
## prazer_pos_pausa        1095  0.79  0.74  0.73   0.69 4.22 2.6
## tristeza_pos_pausa      1095  0.75  0.76  0.72   0.65 2.02 2.4
## raiva_pos_pausa         1095  0.70  0.74  0.71   0.61 1.27 2.0
## medo_pos_pausa          1095  0.65  0.69  0.63   0.55 1.35 2.0
## nojo_pos_pausa          1095  0.51  0.58  0.50   0.43 0.54 1.4
```

There were high values of Cronbach's alpha (Pre: $\alpha =$ 0.884; Post Film: $\alpha =$ 0.911; Post Relaxation: $\alpha =$ 0.880), indicating consistency of the questions used to measure intensity of emotions. Therefore, it seems appropriate to use the sum of the items as an Emotion Score (As mentioned above, positive emotion points were inverted before calculating Cronbach's alpha and before generating the Score)

2. Emotional state changes after watching films with positive or negative emotional valence and after a relaxation audio.

2.1. Are participants under social distancing different before our intervention?

Before carrying out further analyses, we checked whether participants that reported following social distancing scored differently than those that did not, before watching the films.

According to the Kolmogorov-Smirnov test, we do not have evidence that the two groups have different scores ($D =$ 0.078; $p =$ 0.494). Accordingly, the data from both groups were aggregated for further analysis.

2.2. Are participants currently working or studying different before our intervention?

Similarly, we evaluated whether participants that were working or studying during the pandemic scored differently than those that were not, before watching the films.

According to the Kolmogorov-Smirnov test, there were differences between the two groups ($D =$ 0.120; $p =$ 0.035) Therefore, we ran a proportion test to check whether the Positive and Negative groups had different proportions of working/studying participants

The proportion test indicated that the proportions (Positive Video Group: 83.7%; Negative Film Group: 85.8%) did not differ ($\chi^2 =$ 0.728; $p =$ 0.393) indicating this variable should not have a major impact in the comparison between groups. Therefore, only film Valence Group and Time variables were considered in the following analyses.

2.3. Testing ANOVA assumptions

The experiment design had two factors: Short animation film valence (Negative or Positive), and Time (Pre film, Post film, Post relaxation). Short animation film valence group is a between-subjects factor and Time is a within-subjects/repeated measures factor. The most appropriate model is a 2-way mixed ANOVA. To run a 2-way mixed ANOVA, we have to check for ANOVA assumptions, namely absence of multicollinearity, normality, homoscedasticity and sphericity. As dependent variable, we are using the Score given by the sum of all responses to the questionnaire, reversing scores for Happiness/Joy, Peacefulness/Calm, Satisfaction and Pleasure/Motivation

2.3.1. Absence of Multicollinearity.

To check the multicollinearity, the generalized variance inflation factors (GVIF) were calculated, with function vif, from car package (https://cran.r-project.org/package=car).

Table ST1: Evaluation of Multicollinearity

	$GVIF$[a]	Df[b]	$GVIF^{(1/(2*Df))}$

[a] Generalized Variance Inflation Factors

[b] Degrees of freedom

	$GVIF$[a]	Df[b]	$GVIF^{(1/(2*Df))}$
Film Valence Group	1.33	1	1.15
Time	3.99	2	1.41
Film Valence Group:Time	4.65	2	1.47

[a] Generalized Variance Inflation Factors

[b] Degrees of freedom

All are relatively low Fox and Monette, 1992 (https://doi.org/10.2307/2290467), therefore there was no indication of multicollinearity.

2.3.2. Normality

To check the normality assumption, we are using the Shapiro-Wilk test as well as generating QQ-plots.

Table ST2: Shapiro-Wilk Normality tests for the total Emotional Intensity score of each subgroup

Film Valence Group	Time	statistic	p
Negative	Pre	0.96	3.010188e-10 $****$
Negative	Post film	0.98	1.754806e-06 $****$
Negative	Post relaxation	0.97	2.912207e-09 $****$
Positive	Pre	0.97	1.009599e-09 $****$
Positive	Post film	0.91	1.017813e-17 $****$
Positive	Post relaxation	0.91	7.013212e-18 $****$

Note:

ns (non-significant, p>=0.05)

$*p$<0.05

$**p$<0.01

$***p$<0.001

$****p$<0.0001

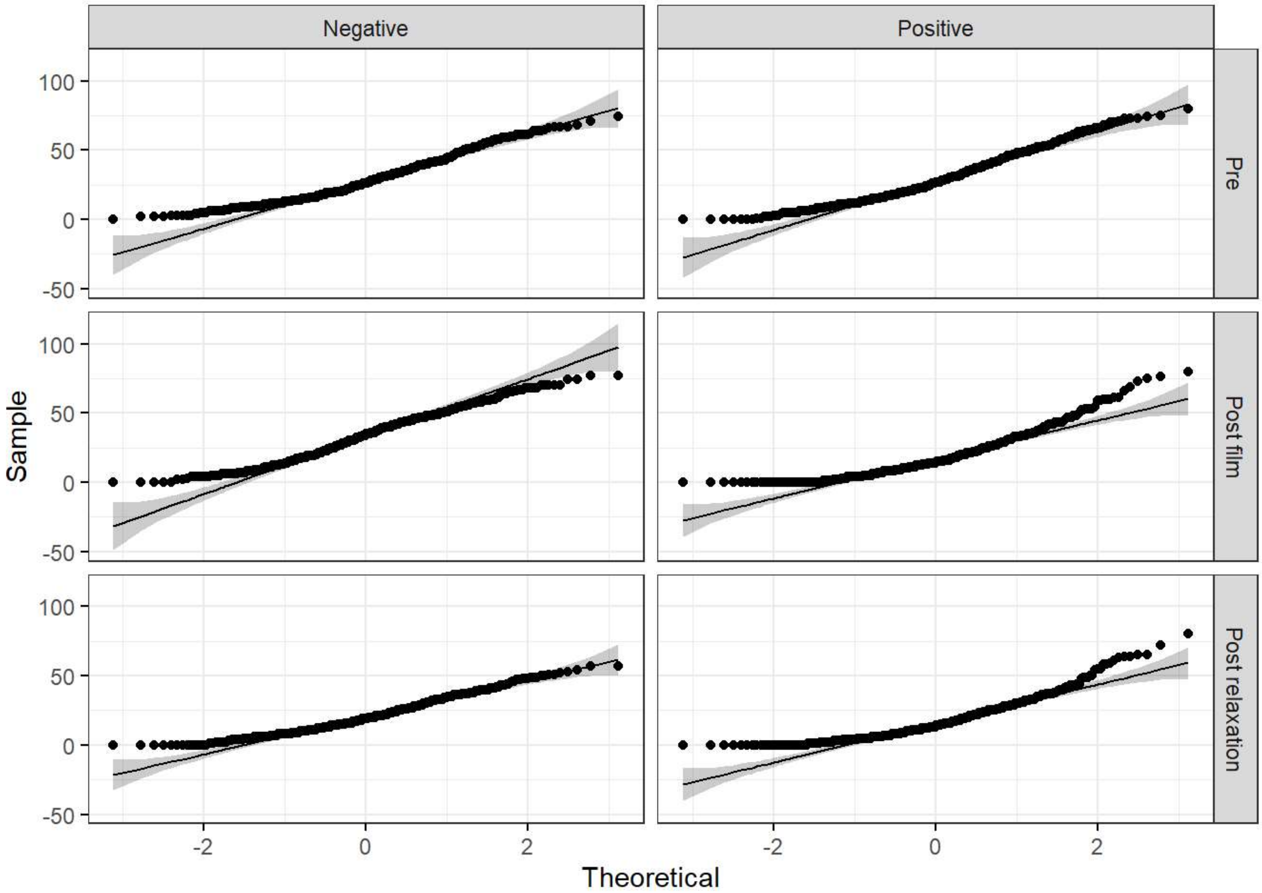

Figure SF1: QQplots to check normality of the Emotional Intensity score.

There is a departure from normality according to the Shapiro-Wilk tests and the QQ plots. Therefore, a method that reduces the impact of violations of ANOVA assumptions is required.

2.3.3. Homoscedasticity

Here we are checking the assumption of homoscedasticity within each within-subject factor levels (Time) using Levene's test.

Table ST3: Levene's tests for the homoscedasticity of the Emotional Intensity score

Time	DFn[a]	DFd[b]	statistic	p
Pre	1	1093	2.85	9.169326e-02 ns
Post film	1	1093	31.33	2.748010e-08 $****$
Post relaxation	1	1093	0.81	3.688767e-01 ns

Note:

ns (non-significant, p>=0.05)

$*p$<0.05

$**p$<0.01

$***p$<0.001

$****p$<0.0001

[a] Numerator degrees of freedom

[b] Denominator degrees of freedom

There was homogeneity of variances for the Pre and the Post relaxation conditions, as assessed by Levene's. However, the Post film condition violated the homogeneity of variance assumption.

2.3.4. Sphericity

The Mauchly's test was used to evaluate sphericity.

There was violation of the sphericity assumption according to the Mauchly's test ($W = 0.86$ $p < 0.0001$).

2.4. Robust ANOVA

Considering the violation of the ANOVA model assumptions, specially the non-normality and the heteroscedasticity of the data, we ran a robust mixed ANOVA using trimmed means using the WRS2 package (Mair and Wilcoxon, 2019) (https://link.springer.com/article/10.3758/s13428-019-01246-w#Sec15).

Table ST4: Trimmed Means ANOVA of the Emotional Intensity score

	Statistic	DFn[a]	DFd[b]	p
Film Valence Group	72.30	1	656.61	1.110223e-16 $****$
Time	377.11	2	511.69	0.000000e+00 $****$
Interaction	160.20	2	511.69	0.000000e+00 $****$

Note:
ns (non-significant, p>=0.05)
$*p$<0.05
$**p$<0.01
$***p$<0.001
$****p$<0.0001
[a] Numerator degrees of freedom
[b] Denominator degrees of freedom

The robust ANOVA revealed significant main effects of Film Valence Group, Time and a significant interaction effect. Considering the limitation of the violation of sphericity, no direct correction was applied. However, the results are extremely significant (p<0.000000000000001), so the increase in Type I error caused by this violation is not enough to explain the observed results.

2.5. Non-Parametric follow-up tests

As follow up tests, non-parametric tests were used. Specifically, a Wilcoxon rank sum test was used to compare film Valence groups, a Friedman test was used to evaluate the main effect of time, and pairwise comparisons using either Wilcoxon rank sum or signed rank when appropriate were used to evaluate the interaction term.

2.5.1. Effect of film valence group (Wilcoxon rank sum test)

Table ST5: Wilcoxon rank sum test as post hoc test of film valence group

Group 1	Group 2	n1	n2	statistic	p	p.signif
Negative	Positive	1644	1641	1674283	4.87e-33	$****$

Note:

ns (non-significant, p>=0.05)

$*p$<0.05

$**p$<0.01

$***p$<0.001

$****p$<0.0001

2.5.2. Effect of time (Friedman test followed by Wilcoxon signed ranked pairwise comparisons)

Table ST6: Time effect with Friedman test

n	statistic	DF[a]	p	
1095	636.45	2	0	$****$

Note:

ns (non-significant, p>=0.05)

$*p$<0.05

$**p$<0.01

$***p$<0.001

$****p$<0.0001

[a] Degrees of freedom

Table ST7: Wilcoxon signed rank tests comparing times

Time 1	Time 2	n1	n2	statistic	corrected p	
Pre	Post film	1095	1095	355606.0	1.49e-15	$****$
Pre	Post relaxation	1095	1095	506740.5	0.00e+00	$****$
Post film	Post relaxation	1095	1095	410007.5	1.11e-62	$****$

Note:

ns (non-significant, p>=0.05)

$*p$<0.05

$**p$<0.01

$***p$<0.001

$****p$<0.0001

[a] Numerator degrees of freedom

[b] Denominator degrees of freedom

2.5.3. Interaction (Wilcoxon signed rank and rank sum pairwise comparisons)

Table ST8: Wilcoxon rank sum tests between Film Valence Groups in each Time

Time	Group 1	Group 2	n1	n2	statistic	corrected p	
Pre	Negative	Positive	548	547	148693.5	1	ns
Post film	Negative	Positive	548	547	227515.0	0	$****$
Post relaxation	Negative	Positive	548	547	180321.0	0	$****$

Note:

ns (non-significant, p>=0.05)

$*p$<0.05

$**p$<0.01

$***p$<0.001

$****p$<0.0001

Table ST9: Wilcoxon signed rank tests comparing Times in each film valence group

Film Valence Group	Group 1	Group 2	n1	n2	statistic	corrected p	
Negative	Pre	Post film	548	548	51956.5	7.860e-07	$****$
Negative	Pre	Post relaxation	548	548	117698.5	9.540e-44	$****$
Negative	Post film	Post relaxation	548	548	127244.0	7.080e-65	$****$
Positive	Pre	Post film	547	547	128654.5	6.600e-71	$****$
Positive	Pre	Post relaxation	547	547	134473.5	4.380e-72	$****$
Positive	Post film	Post relaxation	547	547	70624.0	1.812e-03	$**$

Note:

ns (non-significant, p>=0.05)

$*p$<0.05

$**p$<0.01

$***p$<0.001

$****p$<0.0001

The results indicate that there was no difference between groups at Pre moment. However the difference was significant Post film and Post relaxation (although with a smaller effect than Post film). In each group, all differences were significant when comparing times (Pre, Post film and Post relaxation). The effect of the relaxation seems smaller in the positive group (possible ground effect), and the film effect seemed smaller in the negative group (the film may cause some positive effects attenuation the main negative emotional effect). Also, the difference between positive and negative groups seemed smaller Post relaxation than in the Post film.

2.5.4. Film valence group by Time interaction summary

Table ST10: Summary statistics for each Film Valence Group in each Time

Film Valence Group	Time	n	min	max	median	q1[a]	q3[b]	iqr[c]	mad[d]	mean	sd	se	ci[e]

Film Valence Group	Time	n	min	max	median	q1[a]	q3[b]	iqr[c]	mad[d]	mean	sd	se	ci[e]
Negative	Pre	548	0	74	26	16	39	23	16.31	28.39	15.38	0.66	1.29
Negative	Post film	548	0	77	34	19	47	28	20.02	33.60	17.32	0.74	1.45
Negative	Post relaxation	548	0	57	19	11	29	18	13.34	20.58	12.29	0.52	1.03
Positive	Pre	547	0	80	27	16	40	24	17.79	28.87	16.65	0.71	1.40
Positive	Post film	547	0	80	14	7	26	19	13.34	17.92	14.82	0.63	1.25
Positive	Post relaxation	547	0	80	13	6	25	19	11.86	16.94	13.70	0.59	1.15

[a] first quartile

[b] third quartile

[c] interquartile range

[d] median absolute deviation

[e] Margin of error based on the 95 percent confidence interval

3. Association between participants' baseline score on the emotional state scale and the possible change of their main emotion after watching films with positive or negative content.

According to the Refractory Period Hypothesis, individuals under a strong emotion could enter the refractory period, in which they are less affected to events that could modulate their current emotional state. Under this hypothesis, in the present study, it could be hypothesized that individuals that tend to maintain the emotion that most affects them before and after the film (are less affected by the film) would be the ones that present more extreme values in the emotional score. To check the relation between the emotional score and emotional change after the film, we used 2-way robust iterated re-weighted least squares (IWLS) ANOVA with Total Score before the film as dependent variable. As independent variables, the model used a variable indicating whether the volunteer had the same main emotion before and after the film and a variable indicating the valence of the main emotion before the film (Positive or Negative). The model considered the variables main effects and also their interaction. The interaction effect is the one associated with the Refractory Period Hypothesis. Here we used the robust method implemented in MASS package, as it allows for more complex models, and we wanted to evaluate possible effects of sociodemographic variables as well.

3.1. Plots to check model assumptions

Here we generated plots to evaluate assumptions of the ANOVA model. In this case, we want to evaluate: multicollinearity, normality and homoscedasticity.

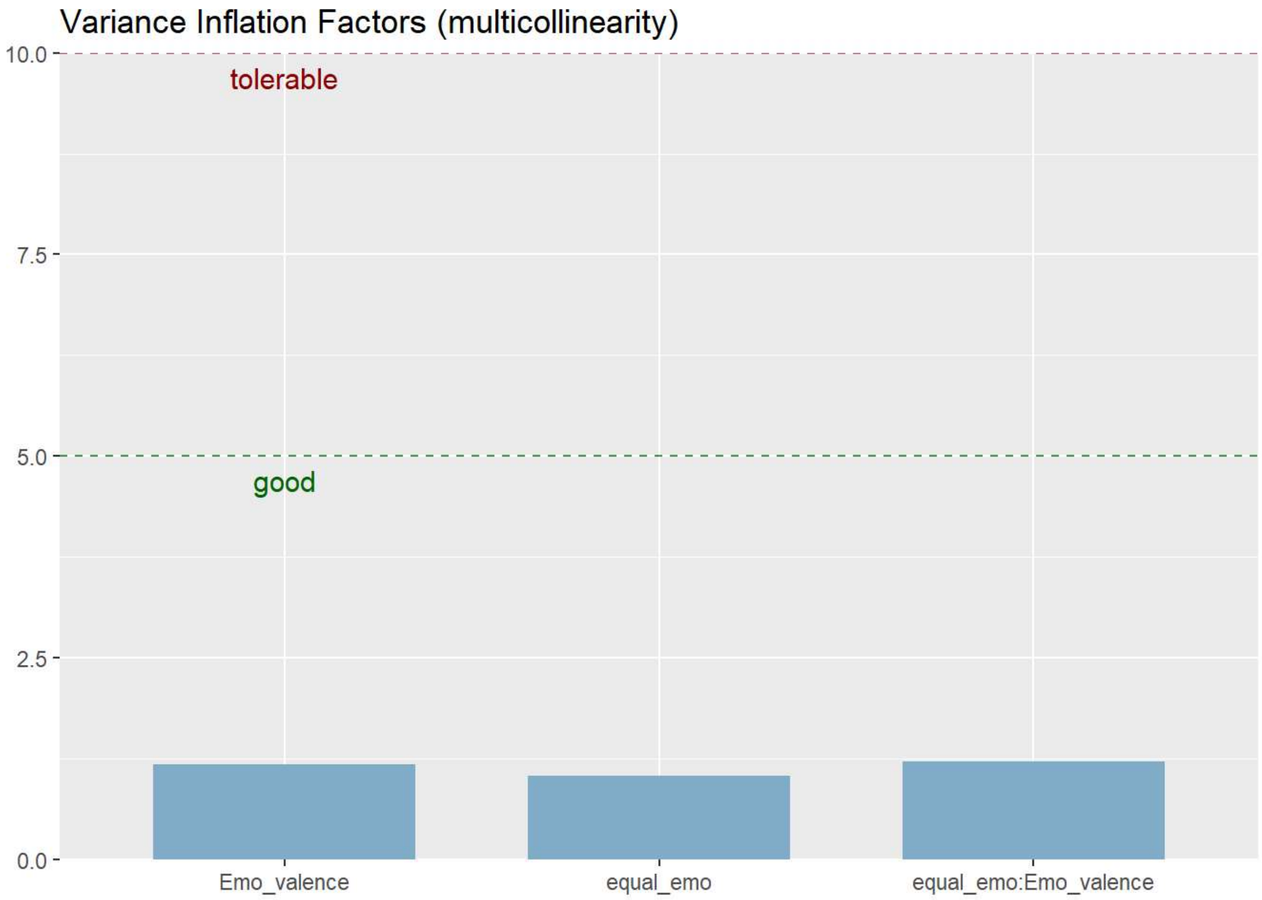

Figure SF2: Assessment of multicolinearity based on Variance Inflation Factors.

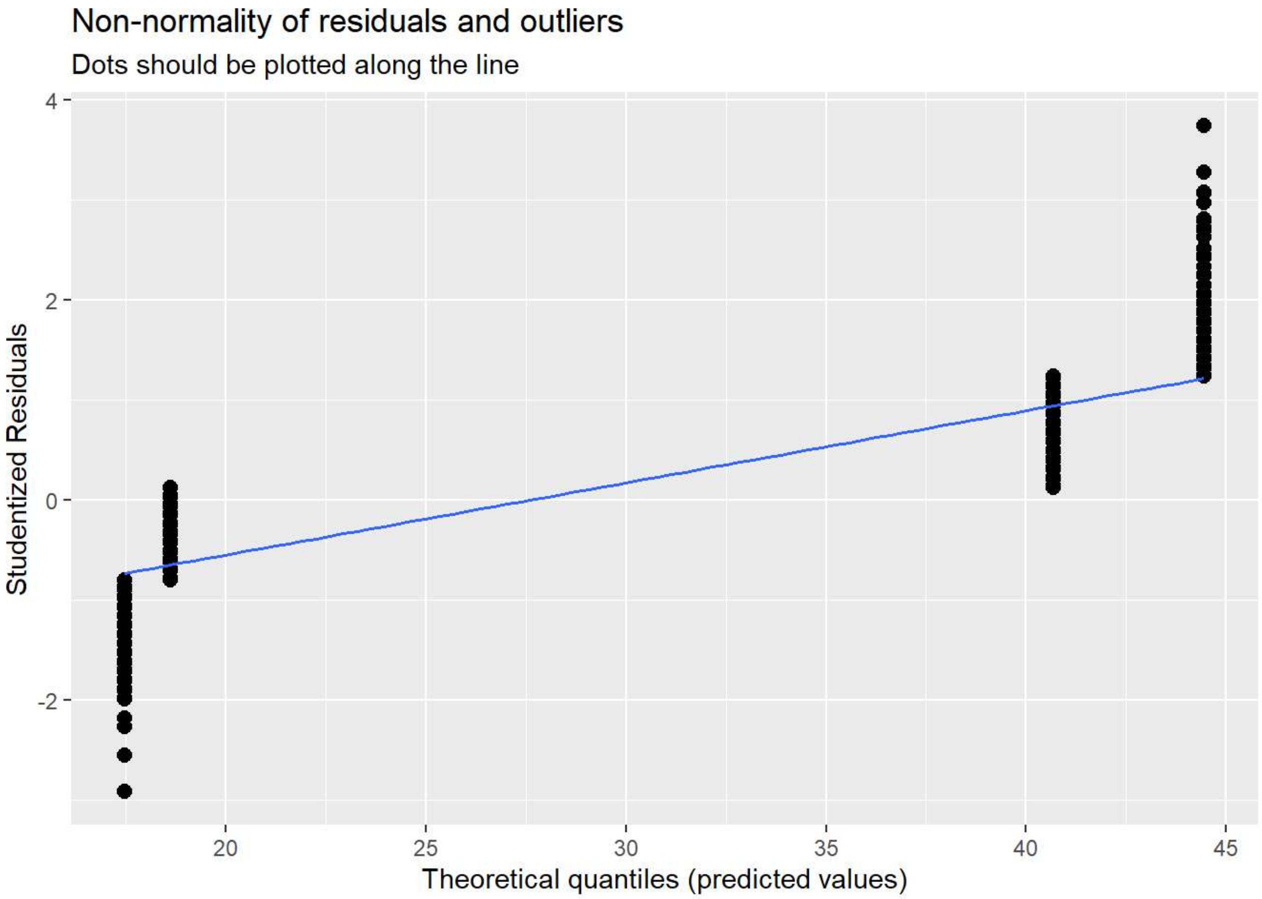

Figure SF3: QQplot to check normality of residuals of the model.

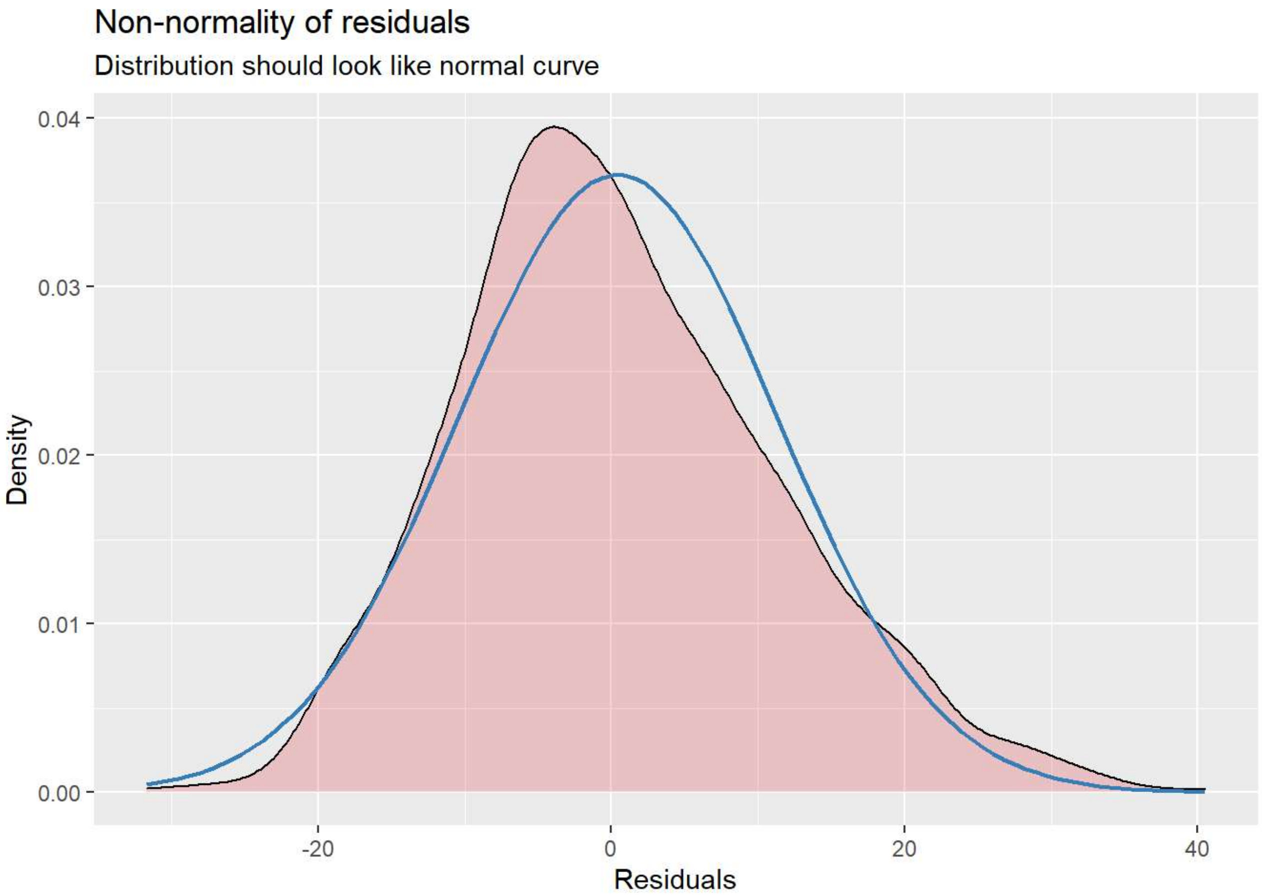

Figure SF4: Plot of the distribuition of residuals to check normality assumption.

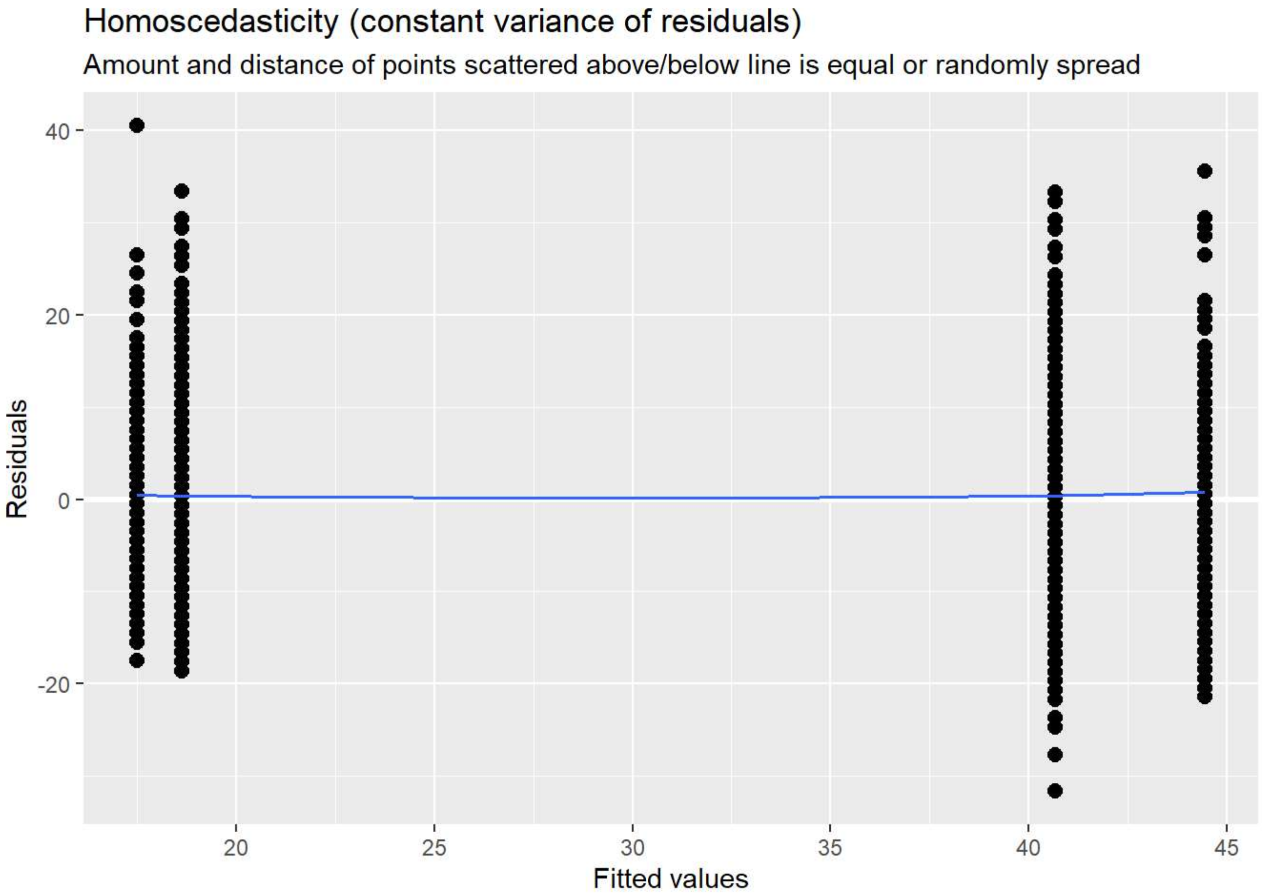

Figure SF5: Plot of the residulas against fittet values to check normality homoscedasticity assumption.

The plots indicate that the only assumption that seems to be violated is normality. To mitigate effects caused by violation of this assumption, two robust methods were adopted: Iterated re-Weighted Least Squares (IWLS) and Trimmed means. IWLS offers the possibility of adding the sociodemographic variables to the model as well. As trimmed means method was used in the previous analysis, We decided to also use it here to confirm the results using different models.

3.2. IWLS Anova Results

Table ST11: IWLS ANOVA evaluating Emotion variation groups and initial emotional valence

Effect	DFn[c]	DFd[d]	F	p
Group[a]	1	1091	3.27	0.071000 ns
Valence[b]	1	1091	1136.44	0.000000 $****$
Group and Valence Interaction	1	1091	11.34	0.000786 $***$

Note:
ns (non-significant, p>=0.05)
$*p$<0.05
$**p$<0.01
$***p$<0.001
$****p$<0.0001
[a] Keep or Change Group
[b] Positive or Negative valence of the main emotion before watching the film
[c] Numerator degrees of freedom
[d] Denominator degrees of freedom

3.3. Trimmed means ANOVA Results

As the Trimmed Means Robust ANOVA was used in other analyses in this study, we also used it here to compare it with the MASS IWLS robust model.

Table ST12: Trimmed Means ANOVA of the Emotional Intensity score at baseline

	Statistic	p
Group[a]	1.62	0.205 ns

Note:
ns (non-significant, p>=0.05)
$*p$<0.05
$**p$<0.01
$***p$<0.001
$****p$<0.0001
[a] Keep or Change Group
[b] Positive or Negative valence of the main emotion before watching the film

	Statistic	p	
Valence[b]	783.12	0.001	$***$
Interaction	8.98	0.004	$***$

Note:
ns (non-significant, p>=0.05)
$*p$<0.05
$**p$<0.01
$***p$<0.001
$****p$<0.0001
[a] Keep or Change Group
[b] Positive or Negative valence of the main emotion before watching the film

In both models, the interaction effect is significant, being in agreement with the study hypothesis. Also, the valence of the emotion has a significant effect, which is expected as negative emotions are presented as higher score values (positive emotions as lower values).

3.4. Post Hoc Tests and Summary table

Table ST13: Wilcoxon rank sum tests between Valence groups in each Variation group

Variation Group	Group 1	Group 2	n1	n2	statistic	p	
Change	Negative emotion	Positive emotion	328	429	129891	1.864e-88	$****$
Keep	Negative emotion	Positive emotion	134	204	26198	7.580e-46	$****$

Note:
ns (non-significant, p>=0.05)
$*p$<0.05
$**p$<0.01
$***p$<0.001
$****p$<0.0001

Table ST14: Wilcoxon rank sum tests between Variation groups in each Valence group

Valence Group	Group 1	Group 2	n1	n2	statistic	p	
Negative emotion	Change	Keep	328	134	18373	0.012	$*$

Note:
ns (non-significant, p>=0.05)
$*p$<0.05
$**p$<0.01
$***p$<0.001
$****p$<0.0001

Valence Group	Group 1	Group 2	n1	n2	statistic	p
Positive emotion	Change	Keep	429	204	47312	0.196 ns

Note:
ns (non-significant, p>=0.05)
$*p$<0.05
$**p$<0.01
$***p$<0.001
$****p$<0.0001

Table ST15: Summary statistics for each Emotion Variation Group and each Initial Emotion Valence

Variation Group	Initial Valence	n	min	max	median	q1[a]	q3[b]	iqr[c]	mad[d]	mean	sd	se	ci[e]
Change	Negative emotion	328	9	74	40	33	49	16	11.86	41.11	12.30	0.68	1.34
Change	Positive emotion	429	0	52	18	12	25	13	8.90	18.99	9.60	0.46	0.91
Keep	Negative emotion	134	23	80	43	35	56	20	14.83	45.22	13.02	1.12	2.22
Keep	Positive emotion	204	0	58	16	11	23	12	8.90	17.94	9.45	0.66	1.30

[a] first quartile
[b] third quartile
[c] interquartile range
[d] median absolute deviation
[e] Margin of error based on the 95 percent confidence interval

Results indicate that participants who kept their main emotion had higher scores when under a negative emotion, and smaller scores when under a positive emotion. In other words, Keep participants had a tendency of having more extreme scores. However, this effect seems subtle. Post-hoc tests indicate that the possible refractory period effect was more evident in the participants with negative emotions, as the difference between Keep and Change groups was significant only for participants with negative emotions.

3.5. Robust Anova with sociodemographic variables

To avoid possible effects related to sociodemographic variables, a robust ANOVA including these variables was used. Were used as sociodemographic variables: gender, age, education level, marital status, use of medication, psychiatric disorder, currently working/studying, following social distancing recommendations, and the score of emotional awareness (sum of 4 questions). The IWLS Robust Anova was used here as it allow for the inclusion of all sociodemographic variables.

Table ST16: IWLS ANOVA evaluating Emotion variation groups and initial emotional valence, including sociodemographic variables.

	DFn[d]	F	p	
Group[a]	1	5.77	1.649899e-02	$*$
Valence[b]	1	1010.37	0.000000e+00	$****$
Interaction[c]	1	4.72	2.998049e-02	$*$
Age	1	0.77	3.801245e-01	ns
Psychiatric Disorders	1	36.32	2.294223e-09	$****$
Medication	1	0.01	9.195616e-01	ns
Education Level	3	3.30	1.989589e-02	$*$
Marital status	4	3.41	8.750281e-03	$**$
Currently working/studying	1	1.49	2.220126e-01	ns
Awareness of emotions	1	67.97	4.807982e-16	$****$
Gender	3	0.18	9.102103e-01	ns
Social Distancing	1	1.22	2.705069e-01	ns

Note:
ns (non-significant, p>=0.05)
$*p$<0.05
$**p$<0.01
$***p$<0.001
$****p$<0.0001
[a] Keep or Change Group
[b] Positive or Negative valence of the main emotion before watching the film
[c] Interaction between Group and Valence
[d] Numerator degrees of freedom

The interaction effect associated with refractory period hypothesis is still significant in this model. In other words, Keep participants had a tendency of having more extreme scores even when controlling for possible sociodemographic effects.

4. The association between the initial main emotion valence and their potential change in this emotion with participants' demographic information.

After observing the interaction effect compatible with the refractory period hypothesis, we decided to evaluate the possible relation of each sociodemographic variables with variation and valence of emotions. For this, 2-way models using each sociodemographic variable as dependent variable, and variation and valence of emotions as independent variables, including their interaction. Different analyses were performed according to the dependent variable type

4.1. Continuous variables

We analyzed continuous variables with a 2-way trimmed means robust ANOVA (Mair and Wilcoxon, 2019) (https://link.springer.com/article/10.3758/s13428-019-01246-w#Sec12)

4.1.1. Age

Table ST17: Trimmed Means ANOVA of Age by emotion variation groups and initial emotional valence

	Statistic	p
Group[a]	4.49	0.035 $*$
Valence[b]	41.48	0.001 $***$
Interaction	4.21	0.041 $*$

Note:

ns (non-significant, p>=0.05)

$*p$<0.05

$**p$<0.01

$***p$<0.001

$****p$<0.0001

[a] Keep or Change Group

[b] Positive or Negative valence of the main emotion before watching the film

Results indicate all effects are significant. It seems that positive groups had higher ages, and that this was observed especially in Keep Positive group.

4.1.2. Awareness of emotions

Table ST18: Trimmed Means ANOVA of Aweareness of emotions by emotion variation groups and initial emotional valence

	Statistic	p
Group[a]	1.59	0.209 ns
Valence[b]	13.66	0.001 $***$
Interaction	1.77	0.185 ns

Note:

ns (non-significant, p>=0.05)

$*p$<0.05

$**p$<0.01

$***p$<0.001

$****p$<0.0001

[a] Keep or Change Group

[b] Positive or Negative valence of the main emotion before watching the film

Awareness was had a tendency to be higher in participants with positive emotions

4.2. Ordinal variables

Ordinal logistic regression models using Variation of Emotion and Emotional Valence as factors, as well as their interaction. To test the significance of each factor, a likelihood ratio Anova was calculated (Analysis of deviance). The only variable analyzed as ordinal was Education Level.

4.2.1. Education Level

Table ST19: Education level ordinal logistic regression by emotion variation groups and initial emotional valence

	Chi-Squared	DFn[c]	p	
Group[a]	0.64	1	4.247500e-01	ns
Valence[b]	16.06	1	6.142431e-05	$****$
Interaction	2.56	1	1.098195e-01	ns

Note:

ns (non-significant, p>=0.05)

$*p$<0.05

$**p$<0.01

$***p$<0.001

$****p$<0.0001

[a] Keep or Change Group

[b] Positive or Negative valence of the main emotion before watching the film

[c] Numerator degrees of freedom

Education level had also a significant effect of emotional valence, suggesting higher education levels were related to a positive emotion.

4.3. Nominal Variables with more than two classes/levels

For nominal variables, multinomial logistic regression models were calculated, using Variation of Emotion and Emotional Valence as factors, as well as their interaction. To test the significance of each factor, a likelihood ratio Anova was calculated (Analysis of deviance). The only variable analyzed as multinomial with more than two classes was Marital status (gender could be considered a multinomial variables, but only 8 participants were not from female or male genders, and we decided to treat gender as a binary variable)

4.3.1. Marital status

```
## # weights:  25 (16 variable)
## initial  value 1762.334514
## iter  10 value 1275.366592
## iter  20 value 1214.577015
## final  value 1213.992878
## converged
```

Table ST20: Marital status multinomial logistic regression by emotion variation groups and initial emotional valence

	Chi-Squared	DFn[c]	p
Group[a]	4.88	4	0.299750651 ns
Valence[b]	20.36	4	0.000424664 $***$
Interaction	7.36	4	0.117815431 ns

Note:

ns (non-significant, p>=0.05)

$*p$<0.05

$**p$<0.01

$***p$<0.001

$****p$<0.0001

[a] Keep or Change Group

[b] Positive or Negative valence of the main emotion before watching the film

[c] Numerator degrees of freedom

Marital status had significant emotional valence effect. This seems related to more negative emotions among singles (higher positivive emotions among married participants). This could be associated with a loneliness effect of being single during the pandemic.

4.4. Nominal Variables with two classes (binary)

For nominal variables with two classes, logistic regression models were calculated, using Variation of Emotion and Emotional Valence as factors, as well as their interaction. To test the significance of each factor, a likelihood ratio ANOVA was calculated (Analysis of deviance). Gender was included as a binary variable as only 8 participants had genders different from female or male. These 8 subjects were only excluded from the gender analysis, and were included in all other analyses (analyses with other sociodemographic variables).

4.4.1. Psychiatric disorders

Table ST21: Psychiatric disorders logistic regression by emotion variation groups and initial emotional valence

	Chi-Squared	DFn[c]	p

	Chi-Squared	DFn[c]	p
Group[a]	1.08	1	2.986069e-01 ns
Valence[b]	32.12	1	1.449059e-08 $****$
Interaction	2.52	1	1.122849e-01 ns

Note:
ns (non-significant, p>=0.05)
$*p$<0.05
$**p$<0.01
$***p$<0.001
$****p$<0.0001
[a] Keep or Change Group
[b] Positive or Negative valence of the main emotion before watching the film
[c] Numerator degrees of freedom

There was higher presence of psychoatric disorders in the negative emotional valence group

4.4.2. Medication

Table ST22: Medication logistic regression by emotion variation groups and initial emotional valence

	Chi-Squared	DFn[c]	p
Group[a]	0.75	1	3.852144e-01 ns
Valence[b]	15.78	1	7.111075e-05 $****$
Interaction	7.50	1	6.159012e-03 $**$

Note:
ns (non-significant, p>=0.05)
$*p$<0.05
$**p$<0.01
$***p$<0.001
$****p$<0.0001
[a] Keep or Change Group
[b] Positive or Negative valence of the main emotion before watching the film
[c] Numerator degrees of freedom

Medication had significant effect of emotional valence and interaction between valence and variation of emotion. These are apparently caused by a higher presence of participants under medication in Keep Negative group, and smaller presence on the Keep Positive group. Also, negative valence had more participants under medication.

4.4.3. Currently working/studying

Table ST23: Currently working/studying logistic regression by emotion variation groups and initial emotional valence

	Chi-Squared	DFn[c]	p	
Group[a]	0.46	1	0.49835184	ns
Valence[b]	3.15	1	0.07606917	ns
Interaction	0.04	1	0.84086278	ns

Note:
ns (non-significant, p>=0.05)
$*p$<0.05
$**p$<0.01
$***p$<0.001
$****p$<0.0001
[a] Keep or Change Group
[b] Positive or Negative valence of the main emotion before watching the film
[c] Numerator degrees of freedom

There was no significant effect for working/studying

4.4.4. Social Distancing

Table ST24: Social distancing logistic regression by emotion variation groups and initial emotional valence

	Chi-Squared	DFn[c]	p	
Group[a]	4.27	1	0.03877705	$*$
Valence[b]	1.63	1	0.20137109	ns
Interaction	0.99	1	0.31895676	ns

Note:
ns (non-significant, p>=0.05)
$*p$<0.05
$**p$<0.01
$***p$<0.001
$****p$<0.0001
[a] Keep or Change Group
[b] Positive or Negative valence of the main emotion before watching the film
[c] Numerator degrees of freedom

Social distancing was related to emotional variation (there were more participants not following social distancing in Keep groups, especially in positive Keep)

4.4.5. Film Valence Group

Table ST25: Film valence group logistic regression by emotion variation groups and initial emotional valence

	Chi-Squared	DFn[c]	p

	Chi-Squared	DFn[c]	p	
Group[a]	5.63	1	0.017694049	$*$
Valence[b]	0.43	1	0.511944846	ns
Interaction	10.61	1	0.001122061	$**$

Note:
ns (non-significant, p>=0.05)
$*p$<0.05
$**p$<0.01
$***p$<0.001
$****p$<0.0001
[a] Keep or Change Group
[b] Positive or Negative valence of the main emotion before watching the film
[c] Numerator degrees of freedom

Film valence group had significant effect of emotion variation and interaction between valence and variation of emotion. It seems that Positive film group was more present in Change Negative Emotion group and less present in Keep Negative Emotion group. In other words, the positive film had higher chance of changing this negative emotion than the negative film (the negative film had only a slight tendency of a similar effect considering the positive emotion groups, and therefore the effect of emotional variation as also significant)

4.4.6. Gender

Table ST26: Gender logistic regression by emotion variation groups and initial emotional valence

	Chi-Squared	DFn[c]	p	
Group[a]	4.29	1	0.03832497	$*$
Valence[b]	0.09	1	0.76569317	ns
Interaction	0.25	1	0.61930829	ns

Note:
ns (non-significant, p>=0.05)
$*p$<0.05
$**p$<0.01
$***p$<0.001
$****p$<0.0001
[a] Keep or Change Group
[b] Positive or Negative valence of the main emotion before watching the film
[c] Numerator degrees of freedom

Gender had a significant effect of emotion variation, with more female being present in groups who varied their emotion. This analysis was carried with 1087 participants, as 8 participants had reported genders other than female or male (we would not be able to analyze the other genders with only 8 participants).

4.5 Demographic tables according to Valance and Variation of emotion groups

Table ST27: Sociodemographic data grouped by Variation of main emotion (Keep vs. Change)

	Change (N=757)	Keep (N=338)
**Age (years)**		
Mean (SD)	40.0 (14.2)	42.5 (13.5)
Median [Min, Max]	40.0 [18.0, 81.0]	42.0 [18.0, 74.0]
**Gender**		
Female	652 (86.1%)	274 (81.1%)
Male	99 (13.1%)	62 (18.3%)
Other	2 (0.3%)	1 (0.3%)
I'd rather not answer	4 (0.5%)	1 (0.3%)
**Marital Status**		
Married	351 (46.4%)	149 (44.1%)
Divorced	112 (14.8%)	51 (15.1%)
I'd rather not answer	7 (0.9%)	1 (0.3%)
Single	275 (36.3%)	128 (37.9%)
Widow/Widower	12 (1.6%)	9 (2.7%)
**Education**		
Lower Secondary Education (Incomplete or Complete)	3 (0.4%)	2 (0.6%)
Higher Secondary Education (Incomplete or Complete)	87 (11.5%)	37 (10.9%)
Undergraduate / Bachelor (Incomplete or Complete)	274 (36.2%)	130 (38.5%)
Graduate, MBA, MSc, PhD (Incomplete or Complete)	393 (51.9%)	169 (50.0%)
**Is following social distancing recommendations**		
No	76 (10.0%)	52 (15.4%)
Yes	681 (90.0%)	286 (84.6%)
**Is currently Working or Studying**		
No	112 (14.8%)	55 (16.3%)
Yes	645 (85.2%)	283 (83.7%)
**Uses medication**		
No	592 (78.2%)	273 (80.8%)
Yes	165 (21.8%)	65 (19.2%)
**psychiatric**		
No	484 (63.9%)	230 (68.0%)
Yes	273 (36.1%)	108 (32.0%)
**Awareness**		
Mean (SD)	30.7 (6.43)	31.1 (7.28)
Median [Min, Max]	32.0 [0, 40.0]	32.0 [0, 40.0]
**Film Valence Group**		
Negative film	365 (48.2%)	183 (54.1%)

	Change (N=757)	Keep (N=338)
Positive film	392 (51.8%)	155 (45.9%)

Table ST28: Sociodemographic data grouped by Emotional valence groups (Positive emotion vs. Negative emotion)

	Negative emotion (N=462)	Positive emotion (N=633)
**Age (years)**		
Mean (SD)	38.2 (14.1)	42.7 (13.7)
Median [Min, Max]	38.0 [18.0, 76.0]	43.0 [18.0, 81.0]
**Gender**		
Female	395 (85.5%)	531 (83.9%)
Male	67 (14.5%)	94 (14.8%)
Other	0 (0%)	3 (0.5%)
I'd rather not answer	0 (0%)	5 (0.8%)
**Marital Status**		
Married	185 (40.0%)	315 (49.8%)
Divorced	63 (13.6%)	100 (15.8%)
I'd rather not answer	4 (0.9%)	4 (0.6%)
Single	198 (42.9%)	205 (32.4%)
Widow/Widower	12 (2.6%)	9 (1.4%)
**Education**		
Lower Secondary Education (Incomplete or Complete)	1 (0.2%)	4 (0.6%)
Higher Secondary Education (Incomplete or Complete)	65 (14.1%)	59 (9.3%)
Undergraduate / Bachelor (Incomplete or Complete)	188 (40.7%)	216 (34.1%)
Graduate, MBA, MSc, PhD (Incomplete or Complete)	208 (45.0%)	354 (55.9%)
**Is following social distancing recommendations**		
No	48 (10.4%)	80 (12.6%)
Yes	414 (89.6%)	553 (87.4%)
**Is currently Working or Studying**		
No	82 (17.7%)	85 (13.4%)
Yes	380 (82.3%)	548 (86.6%)
**Uses medication**		
No	344 (74.5%)	521 (82.3%)
Yes	118 (25.5%)	112 (17.7%)
**psychiatric**		
No	258 (55.8%)	456 (72.0%)
Yes	204 (44.2%)	177 (28.0%)
**Awareness**		
Mean (SD)	29.7 (7.68)	31.7 (5.75)
Median [Min, Max]	31.0 [0, 40.0]	32.0 [0, 40.0]

	Negative emotion (N=462)	Positive emotion (N=633)
**Film Valence Group**		
Negative film	225 (48.7%)	323 (51.0%)
Positive film	237 (51.3%)	310 (49.0%)

5. Additional information about main initial emotion and emotion variation

In this section we included detailed numbers about participant's initial prevailing emotion and about the types of variation of this main emotion

5.1 Details about initial prevailing emotion

Of the total 1095 participants, 633 (57.8%) had a positive initial main emotion, while 462 (42.2%) had a negative prevailing emotion before watching the film. Detailed numbers of each emotion are presented in Table ST29 below:

Table ST29: Number of participants by initial prevailing emotion

Initial Emotion	Number of participants	Percentage
Anger	102	9.3
Disgust/Aversion	10	0.9
Fear	116	10.6
Happiness/Joy	79	7.2
Peacefulness/Calm	392	35.8
Pleasure/Motivation	45	4.1
Sadness	234	21.4
Satisfaction	117	10.7

5.2 Details about emotional variation groups

Of the total 1095 participants, 757 (69.1%) changed their main emotion after watching one of the films, while 338 (30.9%) had the same prevailing emotion before and after watching one of the films.

Of those who changed, 414 (54.7%) participants changed their emotion to an emotion with a different valence, and 343 (45.3%) participants changed their emotion to an emotion with the same valence. Detailed numbers of participants according to the main emotion before and after the films are presented in the tables (ST30 and ST31) below:

Table ST30: Number of participants by prevailing emotion pre and post film

Initial Emotion Valence	Emotion Valence after Film	Number of participants	Percentage
Negative emotion	Negative emotion	78	10.3
Negative emotion	Positive emotion	250	33.0

Initial Emotion Valence	Emotion Valence after Film	Number of participants	Percentage
Positive emotion	Negative emotion	164	21.7
Positive emotion	Positive emotion	265	35.0

Table ST31: Number of participants by film valence by prevailing pre and post film

Initial Emotion Valence	Emotion Valence after Film	Film Valence	Number of participants	Percentage
Negative emotion	Negative emotion	Negative	59	7.8
Negative emotion	Negative emotion	Positive	19	2.5
Negative emotion	Positive emotion	Negative	83	11.0
Negative emotion	Positive emotion	Positive	167	22.1
Positive emotion	Negative emotion	Negative	154	20.3
Positive emotion	Negative emotion	Positive	10	1.3
Positive emotion	Positive emotion	Negative	69	9.1
Positive emotion	Positive emotion	Positive	196	25.9
