# Supplementary material for: Baseline emotional state influences on the response to animated short films: A randomized online experiment
Source: Front Psychol. 2022 Dec 15;13:1009429. doi: 10.3389/fpsyg.2022.1009429 (PMC9797995; doi:10.3389/fpsyg.2022.1009429)
Supplement: Supplementary file 3 [file Table_1.DOCX]

**Supplementary Table 1**: Social demographic characteristics of the excluded participants (participants who gave informed consent, were at least 18 years old, and lived in Brazil, but did not complete the all the steps of the experiment).

|  | **Total** |
| --- | --- |
|  | **(N=1044)** |
| **Gender** |  |
| Female | 759 (82.1%) |
| Male | 156 (16.9%) |
| Other | 4 (0.4%) |
| I'd rather not answer | 6 (0.6%) |
| Missing* | 119 (11.4%) |
| **Age (years)** |  |
| Mean (SD) | 38.5 (15.0) |
| Median [Min, Max] | 38.0 [18.0, 81.0] |
| Missing* | 127 (12.2%) |
| **Age groups (years)** |  |
| < 25 | 220 (24.0%) |
| 25-34 | 162 (17.7%) |
| 35-44 | 241 (26.3%) |
| 45-54 | 137 (14.9%) |
| 55-64 | 103 (11.2%) |
| >= 65 | 54 (5.9%) |
| Missing* | 127 (12.2%) |
| **Education** |  |
| Primary Education (Incomplete or Complete) | 4 (0.4%) |
| Lower Secondary Education (Incomplete or Complete) | 5 (0.5%) |
| Higher Secondary Education (Incomplete or Complete) | 142 (15.4%) |
| Undergraduate / Bachelor (Incomplete or Complete) | 351 (38.0%) |
| Graduate, MBA, MSc, PhD (Incomplete or Complete) | 422 (45.7%) |
| Missing* | 120 (11.5%) |
| **Monthly income** (R$)** |  |
| 0,00 - 1.045,00 | 52 (5.6%) |
| 1.045,01 - 3.135,00 | 200 (21.7%) |
| 3.135,01 - 6.270,00 | 185 (20.0%) |
| 6.270,01 - 9.405,00 | 94 (10.2%) |
| 9.405,01 - 12.540,00 | 60 (6.5%) |
| 12.540,01 - 15.675,00 | 42 (4.5%) |
| 15.675,00 or more | 161 (17.4%) |
| I'd rather not answer | 129 (14.0%) |
| Missing* | 121 (11.6%) |
| **Marital Status** |  |
| Married | 403 (43.6%) |
| Divorced | 104 (11.2%) |
| I'd rather not answer | 14 (1.5%) |
| Single | 398 (43.0%) |
| Widow/Widower | 6 (0.6%) |
| Missing* | 119 (11.4%) |
| **Is currently Working or Studying** |  |
| No | 173 (18.7%) |
| Yes | 751 (81.3%) |
| Missing* | 120 (11.5%) |
| **Is following social distancing recommendations** |  |
| No | 149 (16.2%) |
| Yes | 773 (83.8%) |
| Missing* | 122 (11.7%) |
| **Psychiatric Disorders** |  |
| Anxiety | 274 (29.7%) |
| Depression | 149 (16.2%) |
| Bipolar disorder | 26 (2.8%) |
| Other(s) | 35 (3.8%) |
| None | 578 (62.7%) |
| Missing* | 122 (11.7%) |
| **Uses medication** |  |
| No | 750 (81.1%) |
| Yes | 175 (18.9%) |
| Missing* | 119 (11.4%) |
| **Awareness** |  |
| Mean (SD) | 29.6 (7.76) |
| Median [Min, Max] | 31.0 [0, 40.0] |
| Missing* | 266 (25.5%) |
| **Film Valence Group** |  |
| Negative film | 515 (49.3%) |
| Positive film | 529 (50.7%) |

Max: maximum; min: minimum; SD: standard deviation.

*There are different amounts of missing values between the items. Percentages of missing values are presented in relation to the total of responses (Total = 1044); the other percentages are relative to the total of answers for the specific question (varies for each question and can be calculated as Total – Missing for each question).

**Brazilian minimum wage at the time of questionnaire application: R$1,045.00.
